# Critical role of the HDAC6–cortactin axis in human megakaryocyte maturation leading to a proplatelet-formation defect

**DOI:** 10.1038/s41467-017-01690-2

**Published:** 2017-11-27

**Authors:** Kahia Messaoudi, Ashfaq Ali, Rameez Ishaq, Alberta Palazzo, Dominika Sliwa, Olivier Bluteau, Sylvie Souquère, Delphine Muller, Khadija M. Diop, Philippe Rameau, Valérie Lapierre, Jean-Pierre Marolleau, Patrick Matthias, Isabelle Godin, Gérard Pierron, Steven G. Thomas, Stephen P. Watson, Nathalie Droin, William Vainchenker, Isabelle Plo, Hana Raslova, Najet Debili

**Affiliations:** 1 Institut National de la Santé et de la Recherche Médicale, UMR 1170, Equipe labellisée par la Ligue Nationale contre le Cancer, 94805 Villejuif, France; 2 Paris-Saclay University, UMR 1170, 94805 Villejuif, France; 3 Gustave Roussy, 94805 Villejuif, France; 4 Paris7 Diderot University, 75013 Paris, France; 5 CNRS-UMR-9196, Institut Gustave Roussy, 94805 Villejuif, France; 6 Genomic Platform, Institut Gustave Roussy, 94805 Villejuif, France; 7 Gustave Roussy, Integrated Biology Core Facility, 94805 Villejuif, France; 8 Gustave Roussy, Cell Therapy Unit, 94805 Villejuif, France; 9 Clinical Hematology and Cell Therapy Department, Amiens Hospital, UPJV University EA4666, 80054 Amiens, France; 10 Friedrich Miescher Institute for Biomedical Research, 4002 Basel, Switzerland; 11 Institute of Cardiovascular Sciences, The Medical School, University of Birmingham, Edgbaston, Birmingham, B15 2TT UK; 12 Centre of Membrane Proteins and Receptors (COMPARE), Universities of Birmingham and Nottingham, Midlands, UK

## Abstract

Thrombocytopenia is a major side effect of a new class of anticancer agents that target histone deacetylase (HDAC). Their mechanism is poorly understood. Here, we show that HDAC6 inhibition and genetic knockdown lead to a strong decrease in human proplatelet formation (PPF). Unexpectedly, HDAC6 inhibition-induced tubulin hyperacetylation has no effect on PPF. The PPF decrease induced by HDAC6 inhibition is related to cortactin (CTTN) hyperacetylation associated with actin disorganization inducing important changes in the distribution of megakaryocyte (MK) organelles. CTTN silencing in human MKs phenocopies HDAC6 inactivation and knockdown leads to a strong PPF defect. This is rescued by forced expression of a deacetylated CTTN mimetic. Unexpectedly, unlike human-derived MKs, HDAC6 and CTTN are shown to be dispensable for mouse PPF in vitro and platelet production in vivo. Our results highlight an unexpected function of HDAC6–CTTN axis as a positive regulator of human but not mouse MK maturation.

## Abstract (web summary)

Histone deacetylase (HDAC) inhibitors, a class of cancer therapeutics, cause thrombocytopenia via an unknown mechanism. Here, the authors show that HDAC6 inhibition impairs proplatelet formation in human megakaryocytes, and show that this is linked to hyperacetylation of the actin-binding protein cortactin.

## Introduction

Megakaryocytes (MKs) are highly specialized bone marrow cells that give rise to anucleated blood cells known as platelets^1^. MK progenitor proliferation occurs by classical mitosis, which, during MK differentiation, subsequently switches to an endomitotic mode^2–4^. At the end of the endomitotic process, MK cytoplasm matures, leading to increased organelle biosynthesis^5^ and the development of the demarcation membrane system (DMS)^6^. Once a MK matures, the DMS extends to form long pseudopods called proplatelets (PPTs)^6^ that fragment, leading to platelet release in marrow sinusoids or lung circulation.^1^ Platelet production is due to MK fragmentation by a dynamic regulation of cytoplasmic extension, which mainly depends on microtubules (MTs) and actin cytoskeleton. While MT sliding powers PPT elongation, actin cytoskeleleton dynamics is critical for early stages of PPF by regulating DMS formation and actomyosin by controlling cortical contractile forces^7–9^. In addition, actin cytoskeleleton is also important for PPT branching and platelet release amplification^10,11^.

A new class of anticancer agents targeting histone deacetylases (HDACs) induce profound thrombocytopenia^12,13^ by several mechanisms, including a toxic effect on hematopoietic progenitors through reactive oxygen species (ROS) and DNA damage and an alteration in late MK differentiation leading to a defect in PPF^14^. The focus has been on the changes in the MK cytoskeleton, especially on tubulin hyperacetylation and MT dynamics alteration as the mechanism of HDACi-induced thrombocytopenia. However, there is no direct evidence that tubulin hyperacetylation is involved in the defect of MK maturation^15^.

In humans, 18 HDACs are grouped into four classes, but the precise role of these proteins in hemostasis is not well defined. HDAC6 belongs to the class IIb of HDACs that shuttles between the cytoplasm and the nucleus^16^. In contrast to class I HDACs, the role of HDAC6 has not yet been described during human megakaryopoiesis. HDAC6 is well expressed in platelets and may be involved in platelet functions^17,18^. Predominantly cytoplasmic^19,20^, HDAC6 possesses two catalytically active domains that deacetylate nonhistone proteins such as tubulin, HSP90, and cortactin (CTTN)^21–23^. While HDAC6 overexpression in diverse cell types results in MT deacetylation, its inhibition induces MT hyperacetylation, which is thought to enhance their stability^23^. Recently, it was shown that *hdac6*-deficient mouse platelets, with hyperacetylated marginal bands, spread faster than their wild-type (WT) counterparts^17,18^. However, the precise role of tubulin hyperacetylation has been the matter of controversy. Indeed, despite tubulin hyperacetylation, *hdac6* knockout mice are viable and develop normally^24^.

Our present work shows that human HDAC6 is a positive regulator of MK terminal differentiation and consequently of PPT generation. Our findings demonstrate that HDAC6 inhibition induces a defect in the development of DMS and α-granules and actin disorganization, thus impairing PPF. This defect is mediated by CTTN hyperacetylation. We also show that HDAC6 inhibition in humans and the mouse displays divergent effects on MK differentiation due to a differential role of CTTN. Altogether, our results highlight the role of HDAC6–CTTN axis in human MK maturation and point to a previously unknown mechanism underlying the HDACi-induced thrombocytopenia^14^.

## Results

### Expression of HDAC6 increases during MK differentiation

**Fig. 1:**
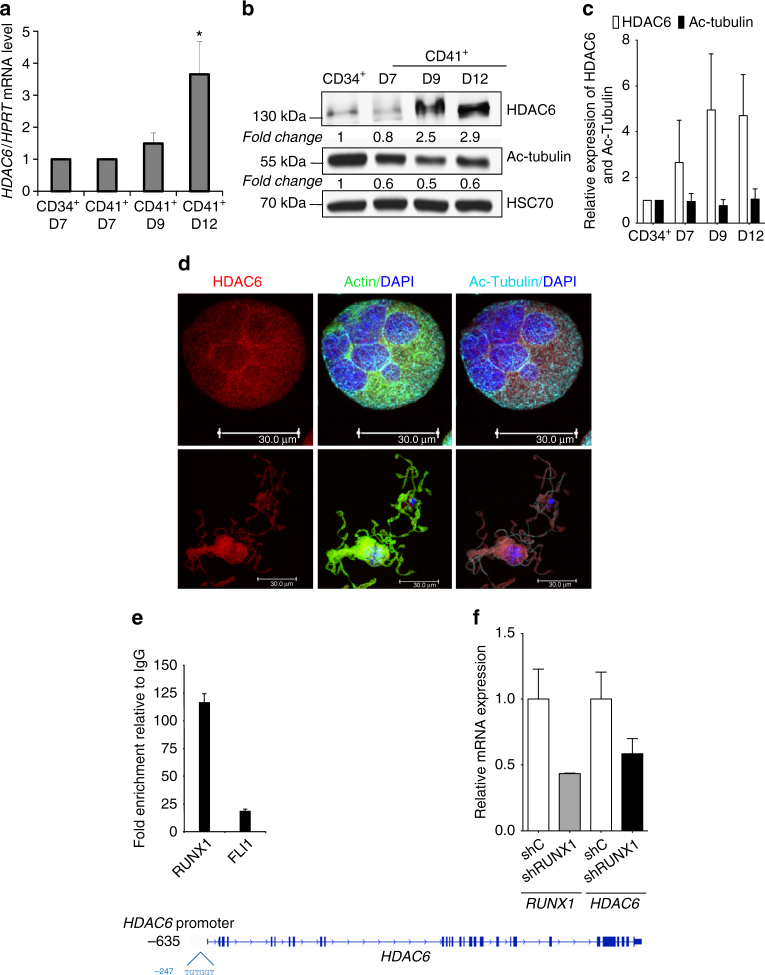
Cellular expression, localization, and regulation of hHDAC6 during human megakaryopoiesis. **a** Relative *HDAC6* mRNA level expression to *HPRT* mRNA at different days of MK maturation evaluated by qRT-PCR. **b**, **c** Quantification of HDAC6 and acetylated tubulin (Ac-Tubulin) protein levels by western blotting during MK differentiation from two independent experiments. HSC70 is used as a loading control. **d** Confocal analysis of HDAC6 (red), actin (green), and ac-Tubulin (cyan) localization at different stages of megakaryopoiesis (D9 MK in the upper panel and PPT in the lower one). **e** The *HDAC6* promoter is regulated by RUNX1 and FLI1 in human MKs. Data are presented as fold enrichment by dividing the % ChIP by % IgG. **f** qRT-PCR analysis of *RUNX1* and *HDAC6* mRNA levels after *RUNX1* knockdown. Error bars in **a**, **e**, and **f** are SD, error bars in **c** are SEM

In order to study the function of HDAC6 during megakaryopoiesis, we determined its expression pattern. CD34^+^ cells were differentiated to MKs and sorted on expression of CD34 and CD41 at day 7 of culture. A fraction of the CD41^+^ cells were grown for 2 and 5 additional days allowing MK maturation. We studied the expression of *HDAC* 1–11 transcripts. *HDAC3* was the HDAC expressed at the highest level all along the MK differentiation and then HDAC2, 1, and 7 (Supplementary Fig. 1). *HDAC6* messenger RNA (mRNA) level was also detected and increased during MK differentiation and was highly expressed at day 12 when MKs were fully mature (Fig. 1a). Similarly, HDAC6 protein was weakly expressed in the CD34^+^ cells and increased along MK maturation to peak at day 12 of culture (threefold increase), as compared to the loading control, HSC70 (Fig. 1b). Concomitantly, acetylated tubulin (ac-tubulin), a major target of HDAC6, was highly expressed in CD34^+^ cells. Tubulin acetylation decreased (about two-fold) during MK commitment and remains stable all along differentiation (Fig. 1b, c). By confocal microscopy, we showed that HDAC6 was predominantly localized in the cytoplasm of MKs and in PPTs where it colocalized with the MT and actin cytoskeleton (Fig. 1d).

### RUNX1 and FLI1 recruitment at the promoter level of HDAC6

To analyze the mechanism of HDAC6 upregulation during megakaryopoiesis, we performed transcription factor-binding site analysis using TRANSFAC (transcription factor database) for the human *HDAC6* promoter. After analysis, we identify a potential RUNX1 site at position –247 and design a qPCR primer set. We next investigated whether RUNX1 might directly associate with the HDAC6 promoter regulatory element in human MKs. To address this question, we performed a chromatin immunoprecipitation (ChIP) study targeting the *HDAC6* promoter area in MKs using antibodies against RUNX1 and FLI1. RUNX1 was highly enriched at the promoter level of *HDAC6* in MKs, whereas a lower enrichment for FLI1 was observed (Fig. 1e). Our data suggest a direct binding of RUNX1 and FLI1 to *HDAC6* promoter close to the transcription start site (TSS) region. To validate these results, we transduced MKs with a shC or a shRUNX1 and found that *RUNX1* knockdown leads to a decrease of HDAC6 expression (Fig. 1f).

### HDAC6 is the main tubulin deacetylase in megakaryocytes

To explore the role of HDAC6 during MK differentiation, two selective HDAC6i, ACY1215 (called ricolinostat) and Tubastatin A, were used. Both inhibitors are widely used to efficiently target HDAC6 without affecting the deacetylase activity of other enzymes at low concentrations^25^^,^^26^.

**Fig. 2:**
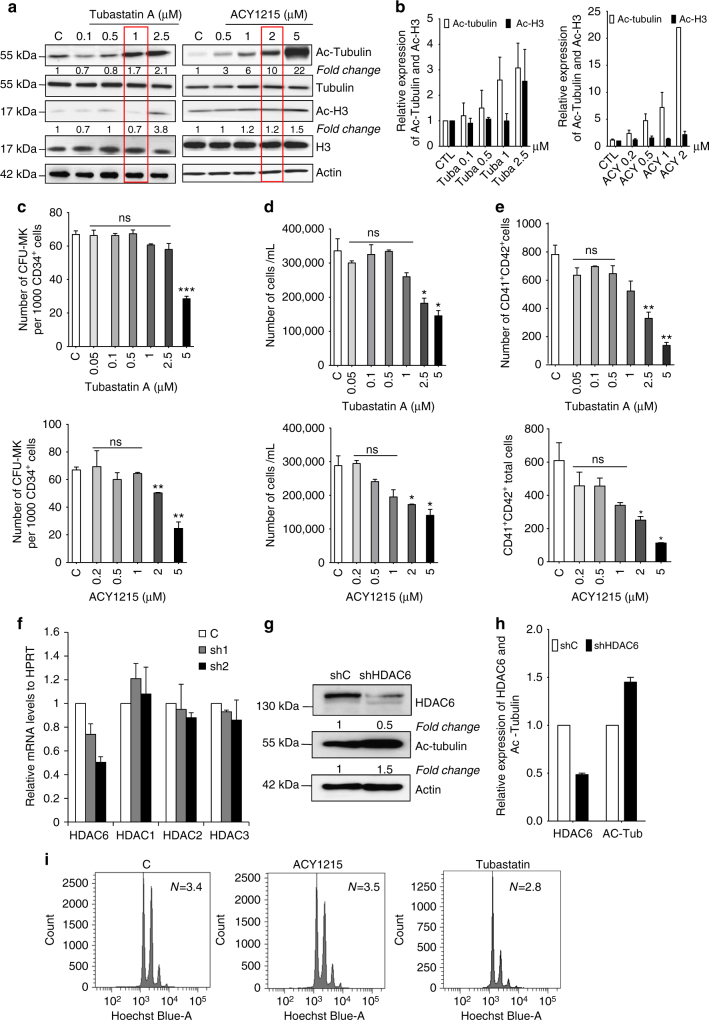
Effects of HDAC6 inhibition on early human megakaryopoiesis. **a**, **b** Effect of increasing doses of HDAC6 inhibitors on the level of Ac-Tubulin and Ac-Histone H3 compared to levels of total Tubulin and Histone H3. **a** Western blot analysis and **b** quantification. **c**–**e** Effect of increasing doses of HDAC6 inhibitors on **c** compartment of CFU-MK, **d** cellular proliferation, and **e** CD41^+^ CD42^+^ population. **f** qRT-PCR analysis of *HDAC6*, *HDAC1, HDAC2*, and *HDAC3* mRNA levels after HDAC6 knockdown with both sh1 and sh2. **g** Western blot analysis of HDAC6 and Ac-Tubulin in sh HDAC6-transduced MKs and **h** quantification from two independent experiments. **i** Mean ploidy value of untreated and Tubastatin- or ACY1215-treated MKs. Results are representative of three independent experiments. Unpaired Student’s *t* test, *p < 0.05; **p < 0.001; ***p < 0.0001. Error bars in **f** are SD, all other error bars are SEM

We first determined their effects on tubulin and histone H3 acetylation by western blot analysis to assess their efficiency and selectivity, respectively. As shown in Fig. 2a, the two inhibitors induced a dose-dependent increase of acetylated α-tubulin, suggesting that HDAC6 might be the main α-tubulin deacetylase in human MKs. As an HDAC6i can, at high concentrations, inhibit other HDAC classes, we take advantage of the fact that in contrast to the other HDACs, HDAC6 does not target H3^25,27,28^. Thus, we considered that the HDAC6-specific concentrations correspond to those that do not alter the level of H3 acetylation. For Tubastatin A, 1 µM was the maximum dose that remains specific for HDAC6 (Fig. 2a). In contrast, ACY1215 begins to induce H3 acetylation at 5 µM (Fig. 2a, b).

It was previously reported that tubulin can also be deacetylated by Sirtuin-2^29^. As can be seen in Supplementary Fig. 2a, Sirtuin2 was expressed during MK differentiation. However, its inhibition did not significantly affect tubulin acetylation level (Supplementary Fig. 2b). Moreover, in the presence of 1 µM ACY1215, a Sirtuin-2 inhibitor, AGK2 did not further increase tubulin acetylation (Supplementary Fig. 2 c, d). Altogether, these results suggest that HDAC6 is the main tubulin deacetylase (TDAC) during megakaryopoiesis.

### HDAC-6 is dispensable for MK progenitors and ploidization

To study whether HDAC6 inhibition may alter the MK progenitor compartment, we measured cell growth with increasing doses of ACY1215 or Tubastatin A (Fig. 2c). At concentrations from 50 nM to 1 µM, no effect on the CFU-MK growth was observed. At higher doses of Tubastatin A and ACY1215, which affect the other HDACs (Fig. 2a), a decrease in CFU-MK growth was observed (Fig. 2c).

To explore if HDAC6 may play a role in proliferation, CD34^+^ cells were seeded in triplicate in serum-free liquid culture in the presence of TPO and increasing doses of ACY1215 or Tubastatin A. Both inhibitors added at day 0 of culture at a concentration of up to 1 µM did not alter the total number of viable cells (Fig. 2d) or the generation of CD41^+^CD42^+^ cells at day 6 of culture (Fig. 2e). Moreover, at 1 µM or less, both inhibitors did not impair the CD34^+^ cell cycle or induce cell death by apoptosis. Only higher doses (from 2 to 5 µM) inhibited cell viability, the total CD41^+^ CD42^+^ cell production (40% inhibition at 2 and 2.5 µM and 75% for 5 µM of both inhibitors), and the percentage of cycling CD34^+^ cells. As these effects on early MK differentiation are only observed at high doses of inhibitors, and mimic the effects of pan-HDACi, these results strongly suggest that this was not related to a selective HDAC6 inhibition. To confirm these pharmacological results, we generated lentiviral constructs encoding two different small hairpin RNA (shRNAs) against *HDAC6* (sh1 and sh2). These constructs, as well as a vector that contained a control sequence (shC), were transduced into differentiating MKs at day 5 of culture. Both shRNAs (sh1 and sh2) efficiently targeted *HDAC6* with a reduction at the mRNA level of 25% for sh1 and 50% for sh2 without affecting *HDAC* 1, 2, and 3 expression levels (Fig. 2f) and led to a 50% decrease at the level of the protein (Fig. 2g). As expected, *HDAC6* knockdown by shRNA increased tubulin acetylation (Fig. 2g, h). Confirming the previous results obtained with chemical inhibitors, the two shRNA did not affect the cell cycle (Supplementary Fig. 3a) or cell viability. This confirms that the effects observed at a low dose of inhibitors are specific of HDAC6 inhibition. Therefore, at a high concentration, both inhibitors have HDAC6-dependent and HDAC6-independent effects, which can be entangled. The effects of HDAC6i on MK polyploidization were mild. While ACY1215 at a concentration of 1 µM or less did not impair MK ploidization, Tubastatin A slightly decreased MK polyploidization, as illustrated by the mean ploidy (CTL *N* = 3.4, ACY1215 *N* = 3.5, and Tubastatin A *N* = 2.8) (Fig. 2i).

### HDAC6 inhibition leads to MK maturation and PPF defects

One critical step for platelet production is MK migration toward the vascular niche along a CXCL12 gradient since PPF and platelet release must occur into the blood circulation. As evidenced in Supplementary Fig. 3b, both drugs led to a moderate, but significant decrease in the number of MK migrating under CXCL12 in vitro (nearly a 20% decrease at 1 µM of both Tubastatin A and ACY1215, *n* = 3, and *p* < 0.05 by unpaired Student’s *t* test). Furthermore, shRNA *HDAC6* inhibition led to a slight decrease in CXCL12-dependent MK migration (Supplementary Fig. 3c).

**Fig. 3:**
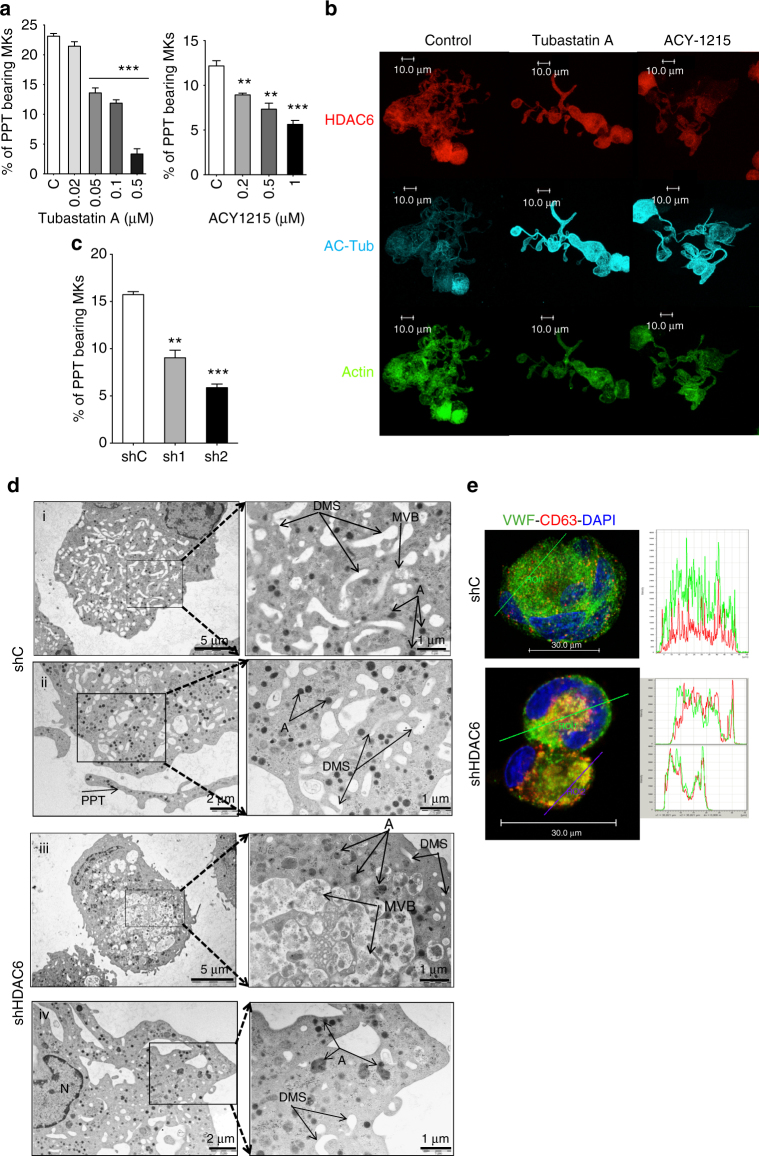
Effects of a pharmaceutical and a genetic inhibition of HDAC6 on terminal human MK differentiation. **a** ACY1215 and Tubastatin A decreases PPF. **b** Confocal analysis of untreated and treated MKs. Proplatelets derived from treated MKs display shorter extensions compared to untreated MKs. Bar represent 10 µm. **c** sh1 and sh2 leads to a specific inhibition of PPF. Results are representative of three independent experiments (*n* = 3). Unpaired Student’s *t* test:**p < 0.001; ***p < 0.0001. **d** Ultrastructure analysis of shC and sh*HDAC6* MKs by transmission electron microscopy. (i, ii) Two MKs with a normal ultrastructure in the sh control with an enlargement, a regular development and distribution of demarcation membrane system (DMS), α-granules (**a**) and multivesicular bodies (MVB). We can also observe normal proplatelet (PPT) produced in vitro. (iii, iv) Two examples of abnormal ultrastructure of sh*HDAC6*-treated MKs. Enlargement on both sh*HDAC6* MKs reveal either (iii) an abnormal presence of cytoplasmic vacuoles containing numerous microvesicles looking to large MVB and giant α-granules, or (iv) mature MKs displaying an heterogeneous granules size content. **e** Confocal analysis of CD63 and vWF in MKs transduced with shC or with sh*HDAC6*. Bar represent 30 µm. Error bars are SEM

To determine if HDAC6 inhibition could alter terminal MK differentiation, we used PPF as a surrogate marker of this process. CD41^+^ cells were sorted on day 8 and further cultured in the presence or absence of HDAC6i. Both inhibitors gave rise to a strong dose-dependent decrease in PPF with around 80% and 60% inhibition, for 500 nM of Tubastatin A and for 1 µM of ACY1215, respectively (Fig. 3a). Higher doses (>1 µM) led to a complete PPF inhibition associated with the presence of apoptotic cells as previously reported^25^. HDAC6i not only altered the rate of PPF, but also induced the generation of shorter and less-branched PPTs (Fig. 3b).

Importantly, sh1 and sh2 against *HDAC6* strongly decreased PPF by 40% and 70%, respectively (*p* < 0.001, *p* < 0.0001) (Fig. 3c). Interestingly, after *HDAC6* knockdown, MKs remained alive and round without forming PPTs, as previously observed with chemical inhibitors.

Ultrastructural studies revealed that *HDAC6* knockdown led to defects of MK maturation characterized by heterogeneity in the DMS and a defect in α-granule development. While the shC-treated MKs showed regular distribution of DMSs and α-granules (Fig. 3d i, ii), the shHDAC6-treated MKs display a paucity in DMSs, the presence of large multivesicular bodies (MVBs), and heterogeneity in the α-granules size with enlarged α-granules (Fig. 3d iii, iv). This result was confirmed by confocal microscopy, showing that shHDAC6-treated MKs display a heterogeneous distribution of granules, as assessed by the presence of the von Willebrand factor (VWF). VWF was colocalized in some granules with the CD63, a lysosomal marker, suggesting that these double-labeled granules were MVBs (Fig. 3e). The results were similar with 1 μM ACY1215 (Supplementary Fig. 4).

### HDAC6 is dispensable for PPF in mice

**Fig. 4:**
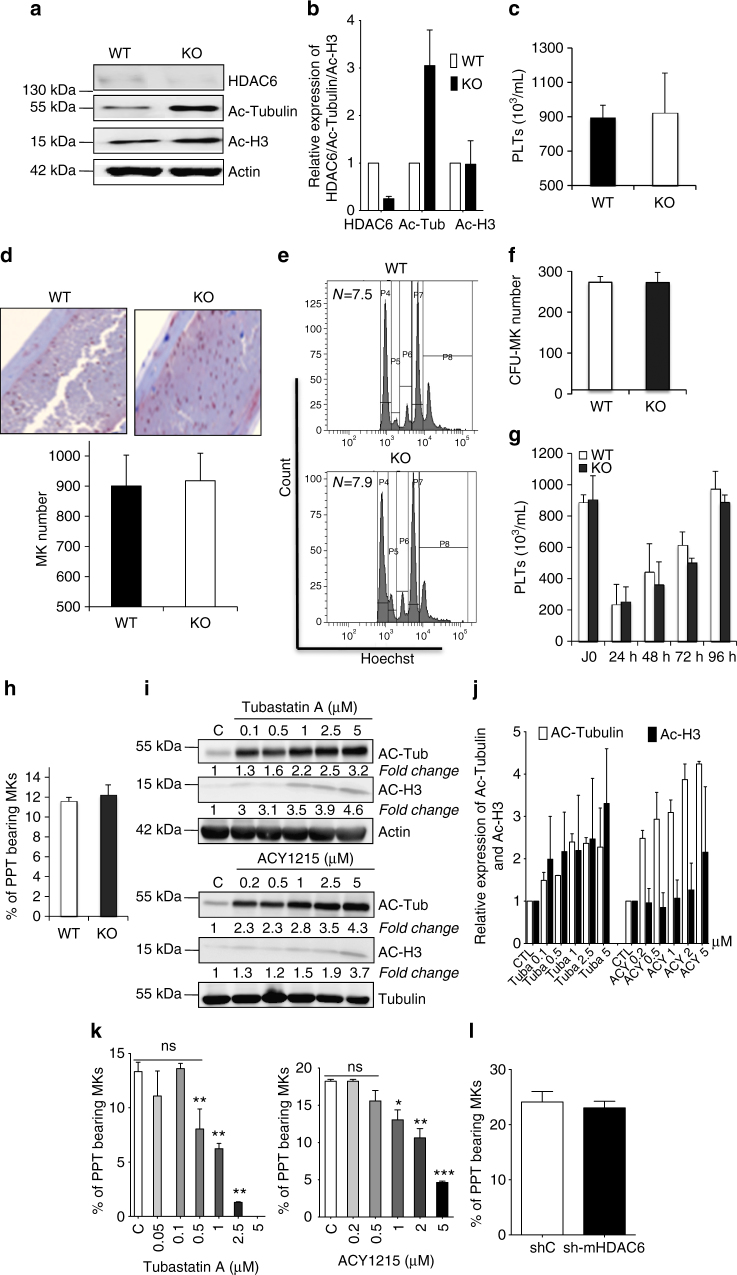
Hdac6 is dispensable for murine megakaryopoiesis. **a**, **b** Western blot analysis and quantification of Hdac6 and acetylated tubulin levels. **c** Platelet count in WT (*n* = 20) and hdac6^−/−^ (*n* = 10) mice. **d** Similar counts of MK number in the WT and Hdac6 KO bone marrows as attested after vWF staining. **e** *Hdac6* knockout does not affect MK polyploidization in vivo. **f** Similar CFU-MK growth in WT and *hdac6* KO mice. **g** Normal recovery of platelet count in WT and KO mice after thrombocytopenia induction by an anti-CD41 antibody. **h** Normal PPF from MKs isolated from WT and *Hdac6* KO bone marrows. **i**, **j**. Western blot analysis and quantification of 48 h-treated MKs with both inhibitors showing a smaller IC50 compared to human MKs and an increase in histones H3 acetylation at 1 μM. **k** In vitro proplatelet formation by MKs treated with Tubastatin A or ACY1215. Unpaired Student’s *t* test, *p < 0.05; **p < 0.001; ***p < 0.0001. **l** A shRNA against murine *hdac6* (sh-mHDAC6) has no effect on PPF in vitro. Results are representative of three independent experiments carried on at least three mice for each group (*n* = 3). Error bars in **b**, **j**, **k**, and **l** are SEM, error bars in **c**, **d**, **f**–**h** are SD

Using constitutive *hdac6* ^–/–^ knockout mice, we next investigated whether Hdac6 affects PPF in the mouse. A complete loss of Hdac6 was associated with a concomitant increase in tubulin acetylation level in BM MKs, which was confirmed by western blot analysis (Fig. 4a, b). Surprisingly, platelet levels (Fig. 4c), MK number (Fig. 4d), ploidy level (Fig. 4e), and CFU-MK number (Fig. 4f) were not affected by *hdac6* deletion. To further confirm that Hdac6 does not play a major role in platelet production, we performed immuno-induced thrombocytopenia by injecting anti-CD41 into WT and *hdac6* ^−/–^ mice. No difference was observed in platelet recovery (Fig. 4g). Moreover, MKs derived from *hdac6* ^–/–^ mice displayed normal PPF in vitro as compared to WT (Fig. 4h).

To characterize whether the absence of a phenotype in the mouse is due to a compensation of the BM microenvironnement or an internal pathway, we investigated the in vitro effects of Hdac6 inhibition on mouse megakaryopoiesis using ACY1215 and Tubastatin A. Both inhibitors induced tubulin α-hyperacetylation in mouse MKs at lower doses (100 and 200 nM, respectively, Fig. 4i) than that which was previously observed for human MKs (Fig. 2a). Interestingly, in the mouse, 1 μM of both inhibitors elicited increased H3 acetylation (Fig. 4i, j). At the Hdac6-specific inhibitory concentrations (<1 μM), cell cycle progression, apoptosis, and endomitosis were not affected as was PPF (Fig. 4k). Moreover, similar to the pharmacological inactivation as well as the genetic ablation, *hdac6* knockdown using shRNA had no obvious effect on PPF in vitro (Fig. 4l). Altogether, these results demonstrate that Hdac6 is dispensable for PPF in mice not only in vivo but also in vitro in contrast to humans.

### HDAC6 localization in human and murine MKs

**Fig. 5:**
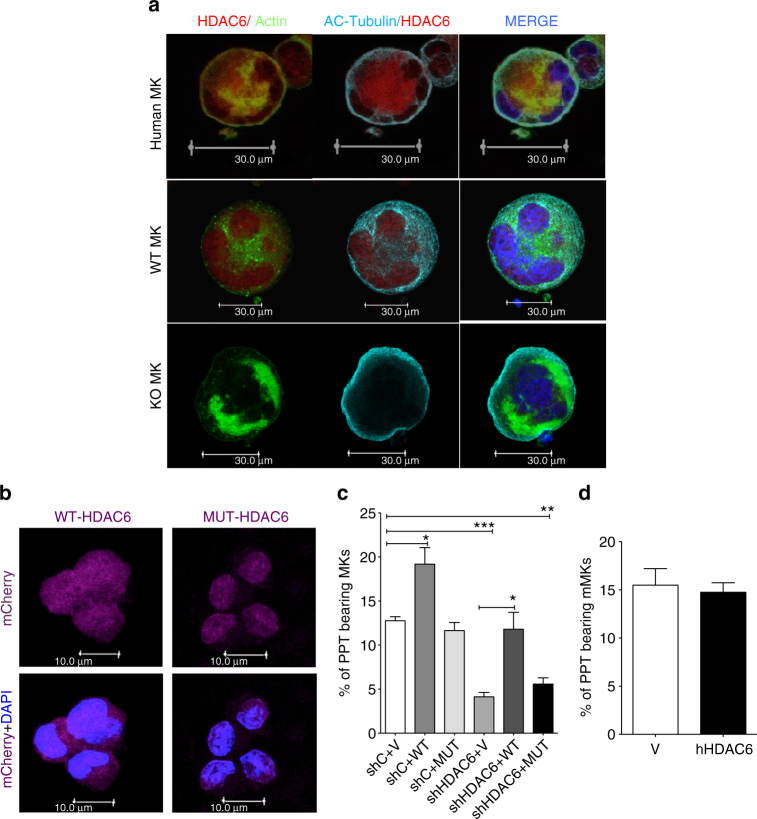
Differential HDAC6 localization in human and murine MKs does not explain the different phenotypes. **a** Immunofluoresence analysis showing localization of mHDAC6 and hHDAC6 in MKs. **b** Immunofluorescence analysis showing cytoplasmic WT-HDAC6 and nuclear MUT-HDAC6 accumulation in human MKs. **c** WT-HDAC6 but not MUT-HDAC6 reversed the shHDAC6-induced PPF decrease in human MKs. hMKs were either cotransduced provide space with sh Control (shC) with our without an empty vector (V) or WT-HDAC6 (cytoplamic) or NES-muted HDAC6 (nuclear, MUT) or cotransduced with shHDAC6 with or without an empty vector or WT-HDAC6 or MUT-HDAC6. **d** Cytoplasmic HDAC6 is not required for murine PPF in vitro. mMKs were transduced with either empty vector (V) or human WT-HDAC6 (hHDAC6, cytoplasmic) and allowed to form PPTs in vitro. Results are representative of three independent experiments. Unpaired Student’s *t* test, *p < 0.05; **p < 0.001; ***p < 0.0001. Error bars are SEM

To better understand these differences, we investigated the subcellular localization of HDAC6 in mouse and human MK. In humans, HDAC6 is predominantly cytoplasmic (Fig. 1d and the first panel of Fig. 5a), whereas murine Hdac6 (mHdac6) was predominantly found in the nucleus with only low cytoplasmic levels (Fig. 5a, middle panel). The specificity of the antibody was confirmed by the absence of staining of *hdac6* ^–/–^ MKs (Fig. 5a, lower panel). ac-tubulin was detected in human and murine MKs. Thus, human and murine HDAC6 have differing cellular localizations, which might explain their functional differences.

### Cytoplasmic HDAC6 regulates PPF in humans, but not in the mouse

It was previously shown that the localization of human (hHDAC6) and mouse Hdac6 (mHdac6) is differently regulated^30^. While mHdacC6 harbors one functional nuclear export signal (NES), hHDAC6 has two functional NES (NES1 and NES2) and a SE14 tetradecapeptide domain, allowing its retention in the cytoplasm^31^. To determine the importance of HDAC6 cellular localization, we used two constructs: WT-HDAC6 sequence (WT-HDAC6) and NES1/2 mutant (MUT-HDAC6) were mutated to prevent recognition by shHDAC6. We show that while WT-HDAC6 was mainly cytoplasmic, MUT-HDAC6 was redistributed in MK nuclei, confirming that NES sequences play a central role in the cytoplasmic retention of human HDAC6 (Fig. 5b). Strikingly, we found that in human MKs, overexpression of WT-HDAC6, but not of MUT-HDAC6, led to a significant increase in PPT counts, confirming the positive role of HDAC6 in MK maturation (Fig. 5c). Moreover, PPF rescue experiments after the introduction of shHDAC6 into MKs show that expression of WT-HDAC6, but not MUT-HDAC6, rescued the HDAC6-induced decrease of PPF (Fig. 5c). These results confirm that the PPF defect after *HDAC6* silencing is specific and the importance of hHDAC6 cytoplasmic retention is probably to deacetylate its cytoplasmic targets.

We then sought to determine whether HDAC6 redistribution in the nucleus of mouse MKs could be at the origin of the absence of a phenotype in *hdac6* ^–/–^ mice. Cytoplasmic WT-HDAC6 was overexpressed in murine MKs. Strikingly, in contrast to humans, WT-HDAC6 expression in mouse MKs did not increase PPT generation (Fig. 5d).

Taken together, this demonstrates that cytoplasmic localization is necessary for human PPF by HDAC6, but unexpectedly, the different localization of human and mouse HDAC6 is not sufficient to explain its differing role in megakaryopoiesis between the species.

### Tubulin hyperacetylation has no impact on PPF

**Fig. 6:**
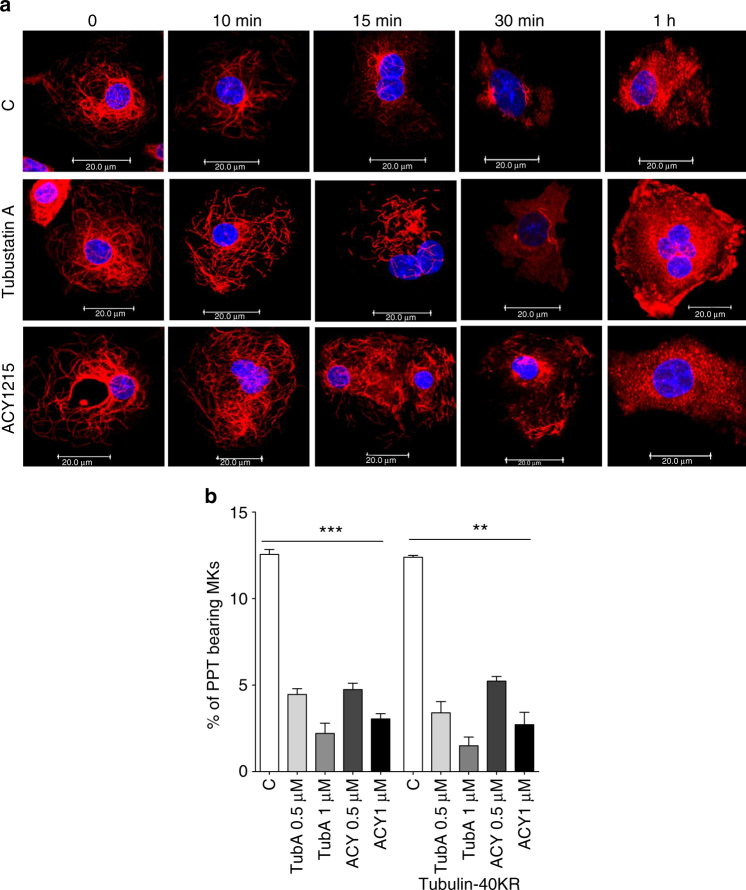
Effects of tubulin acetylation on proplatelet formation. **a** Kinetics of MT depolymerization. CD41^+^ cells treated with either Tubastatin A or ACY1215 were treated with 1 µM Nocodazole for 10, 15, 30 min, and 1 h and stained against β-Tubulin (red) and DAPI (blue). **b** Deacetylated tubulin mimic (Tub-40KR) does not rescue the PPF defect induced by both HDAC6i. (*n* = 4). Unpaired Student’s *t* test **p < 0.001; ***p < 0.0001. Error bars are SEM

We next investigated by which effector(s) HDAC6 modulates MK maturation and PPF. Considering the available data in the literature involving the possible role of ac-tubulin in HDACi-induced thrombocytopenia, we first investigated whether the HDAC6i-induced PPF decrease was due to tubulin hyperacetylation. It was suggested that tubulin hyperacetylation may increase MT stability and rigidity leading to PPF arrest. To test this hypothesis, CD41^+^ cells treated with either Tubastatin A or ACY1215 were incubated at 37 °C in the presence of 1 µM nocodazole to allow MT depolymerization. Quite surprisingly, the kinetics of MT depolymerization in HDAC6i-treated MKs were comparable to that observed in untreated cells (Fig. 6a), indicating that HDAC6i-induced tubulin hyperacetylation does not modify MT stability in MKs.

To assess the role of tubulin hyperacetylation in PPF, we first studied the main tubulin acetyltransferase αTAT expression along MK differentiation. As for ac-tubulin level, αTAT decreased from the CD34 to the CD41 stage and remains stable all along MK differentiation (Supplementary Fig. 5a). In line with our previous observations, αTAT-knockdown (~50% decrease, Supplementary Fig. 5b) resulted in a similar PPF compared to cells transduced with a control vector (Supplementary Fig. 5c), suggesting that the level of ac-tubulin is not a major regulator of PPF.

Interestingly, the overexpression of a non-ac-tubulin construct (Tub-40KR) in human MKs failed to rescue the PPF defect induced by ACY12115 or Tubastatin A (Fig. 6b), suggesting that tubulin acetylation is not the major effector of HDAC6 in its effects of PPF.

### Cortactin is indispensable for MK maturation and PPF

**Fig. 7:**
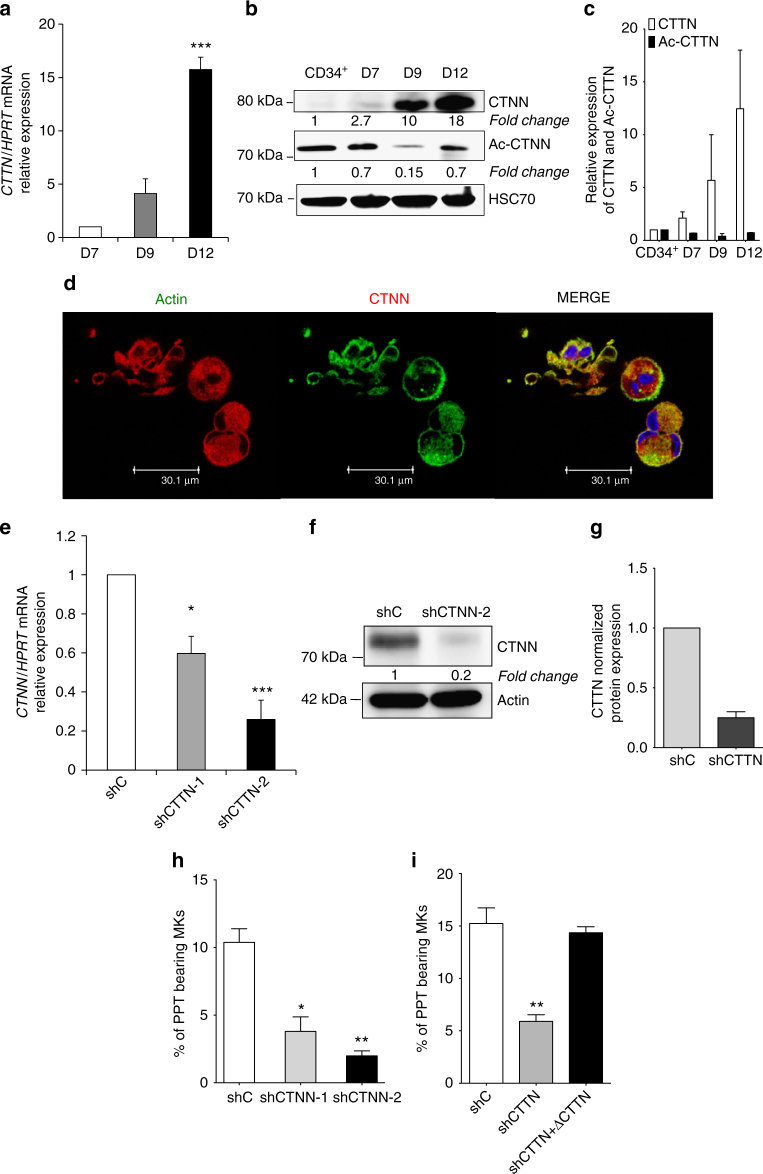
Cortactin is indispensable for human proplatelet formation. **a** Relative *CTTN* mRNA level expression to *HPRT* mRNA at different days of MK maturation. **b**, **c** Western blot analysis and quantification of acetylated cortactin (Ac-CTTN) and total CTTN during MK differentiation. HSC70 is used as a loading control. **d** Confocal analysis of CTTN (red) and actin (green) showing the colocalization of CTNN and actin in MKs and in MKs forming PPF at Day 12 of culture. **e** mRNA level of *CTNN* in shC and shCTNN transduced MKs. **f**, **g** Western blot analysis and quantification showing the decrease of CTTN in shCTTN-treated MKs. **h** *CTTN* knockdown in MKs with shCTTN-1 or shCTTN-2 impairs PPF. **i** Defect of PPF induced by shCTTN is rescued by the reintroduction of CTNN. MKs were transduced as described with either the shC (GFP) or with shCTTN (GFP) and then with silently mutated CTTN (cherry). PPTs were scored at day 12 when MKs are fully mature. Unpaired Student’s *t* test *p < 0.05; **p < 0.001; *p* < 0.0001. Error bars in **a** and **e** are SD, error bars in **c**, **g**–**i** are SEM

Given these results, we centered our investigations on CTTN, another well-characterized HDAC6 target. We first studied CTTN level during normal MK differentiation and showed that CTTN markedly increased (around 15-fold) from day 7 to day 12 of culture both at the level of mRNA and protein (Fig. 7a–c). Confocal imaging revealed a cytoplasmic staining of CTTN that colocalized with the actin cytoskeleton. This was particularly evident at day 12 where the expression of CTTN was high (Fig. 7d). To determine whether CTTN is critical for PPF, we generated two efficient shRNAs targeting *CTTN* (shCTTN-1 and shCTTN-2) that led to 40% and 80% decrease at mRNA and protein level, respectively (Fig. 7e–g). As for *HDAC6* silencing, neither shRNAs affected the cell cycle (Supplementary Fig. 6a) or MK maturation assessed by the expression of the CD41 and CD42 (Supplementary Fig. 6b), but lead to a moderate decrease of the mean ploidy (shC *N* = 4.9, shCTTN *N* = 3.3, Supplementary Fig. 6c) and significantly decreased MK migration (Supplementary Fig. 6d, *p* < 0.05). Strikingly, *CTTN* silencing led to a sharp decrease in PPF compared to cells transduced with a control shRNA (shC) (Fig. 7h, *p* < 0.05 and *p* < 0.01 for shCTTN-1 and shCTTN-2, respectively).

To confirm these results, a CTTN construct with a silent mutation to prevent recognition by the shRNA was expressed in MKs already transduced with shCTTN. Reintroduction of CTTN completely reversed the PPF defect induced by shCTTN, clearly demonstrating that the phenotype of the two shRNAs was not due to off-target effects (Fig. 7i).

### HDAC6i inhibits PPF through cortactin hyperacetylation

**Fig. 8:**
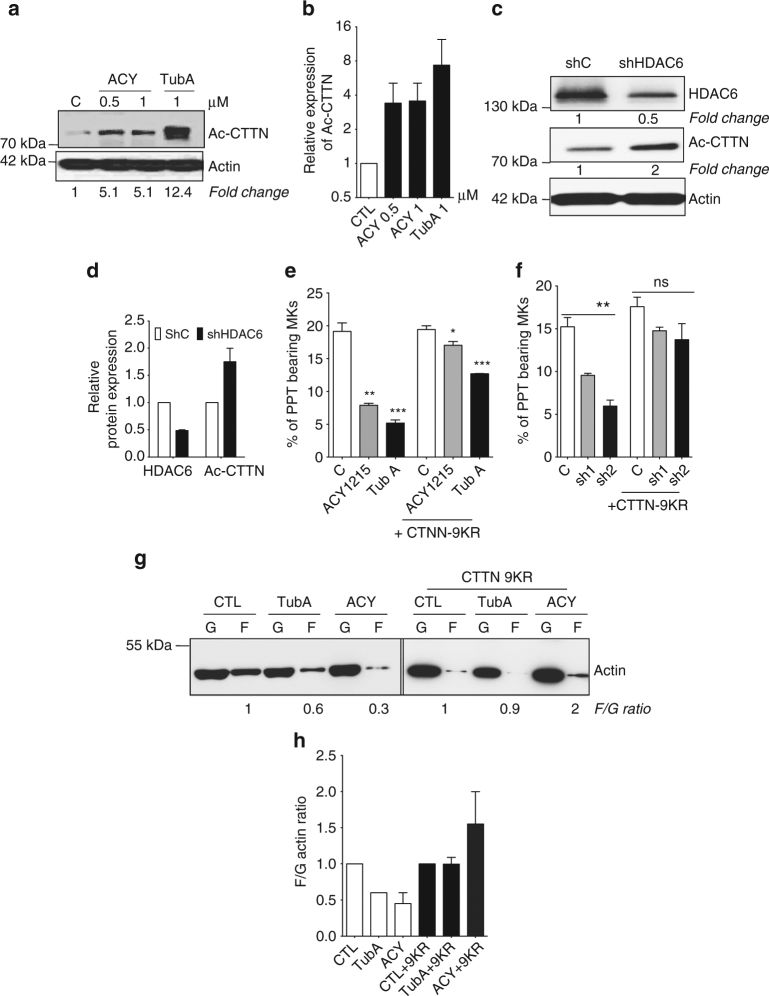
HDAC6i decreases proplatelet formation through CTTN hyperacetylation. **a**, **b** Western blot analysis and quantification showing the hyperacetylation of CTNN in MKs treated for 48 h with HDAC6 inhibitors (ACY1215 or Tubastatin) compared to untreated MKs. **c**, **d** *HDAC6* knockdown with shRNA increases CTTN acetylation level. **e**, **f** Expression of a CTTN-9KR, a non-acetylable form of CTNN reversed the PPF decrease induced by both HDAC6i (**e**) and by shHDAC6 (**f**). Results are representative of three independent experiments (*n* = 3). Unpaired Student’s *t* test *p < 0.05; **p 0.001; ***p < 0.0001. **g**, **h** Hyperacetylated CTTN decreases F-actin in MKs. **g** MKs transduced with either an empty vector or a lentivirus encoding deacetylated CTTN mimic (CTTN-9KR) were sorted, then treated for 48 h with either 1 μM of Tubastatin A or ACY1215. G and F actin were both separated by ultracentrifugation and lysates were analyzed by western blot. **h** Quantification of western blotting experiments from two independent experiments. Error bars are SEM

Interestingly, as for ac-tubulin, CTTN acetylation decreased through maturation to increase slightly at day 12 of differentiation (Fig. 7b). Inhibition of HDAC6 by Tubastatin or ACY1215 led to an increase in CTTN acetylation (Fig. 8a, b). Interestingly, cells treated with Tubastatin A displayed greater hyperacetylation levels than those observed with ACY1215. Moreover, western blot analysis showed that selective *HDAC6* knockdown with shRNA also increased CTTN acetylation (Fig. 8c, d). It was previously reported that both HDAC6 and Sirtuin1 deacetylate CTTN in vitro in a cell-type-dependent manner. Although Sirtuin1 was expressed in MKs (Supplementary Fig. 6e), its inhibition did not decrease in vitro platelets production (Supplementary Fig. 6f).

To determine whether CTTN is the main effector of HDAC6 during PPF, we overexpressed a non-acetylated CTTN (CTTN9KR, substitution of the nine acetylated lysines by arginine) in MKs. It is known that HDAC6 regulates the activity of CTTN by deacetylating these lysines on the F-actin-binding domain. Overexpression of CTTN9KR fully rescued the PPF defect induced by ACY1215, and partially by Tubastatin A (Fig. 8e). To establish more specific targeting of HDAC6, we performed similar experiments with either shHDAC6 and as expected, CTTN-9KR fully rescued the defect of PPF (Fig. 8f).

It is widely accepted that CTTN deacetylation by HDAC6 increases its affinity for F-actin and enhances its polymerization. To gain further insights into the role of HDAC6-CTTN acetylation during PPF, we explored the effects of HDAC6i on actin dynamics. G (globular) and F (filamentous) actin of CD41^+^ cells were separated by ultracentrifugation, as shown in Fig. 8g. Tubastatin A and ACY1215 led to a decrease in F-actin compared to untreated cells. Consistent with our previous findings (Fig. 8e, f), CTTN9KR expression completely rescued the level of F-actin in cells treated with ACY1215 and partially in cells treated with Tubastatin A compared to control cells (Fig. 8g, h). Together, these findings demonstrate that HDAC6 inhibition decreases actin polymerization that is crucial for both normal DMS development^9^ and PPT branching^11^ through CTTN hyperacetylation.

### HDAC6-cortactin pathway has no effect on murine PPF

**Fig. 9:**
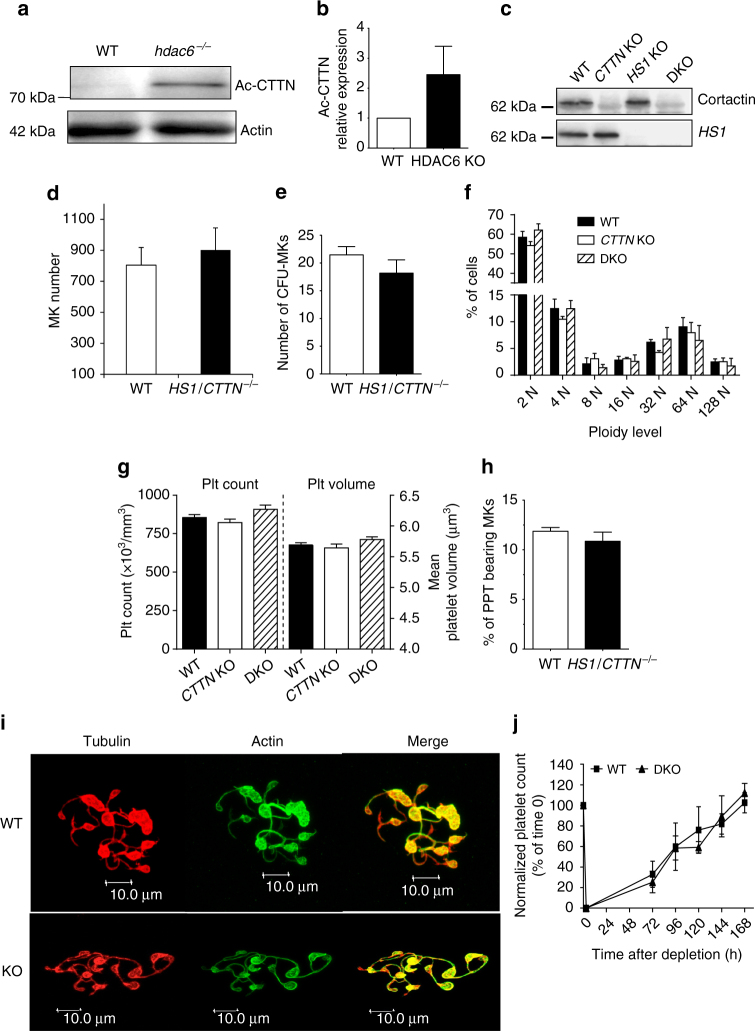
Cortactin is not required for mouse megakaryopoyesis. **a**, **b** *hdac6*-null MKs displayed increased CTTN acetylation compared to WT MK. **c** Platelet extracts from WT, *CTTN* KO, *HS1*, and *HS1/CTTN*(DKO) mice were probed with anti-CTTN or with anti HS1. **d** MK number within bone marrow from WT and *CTTN/HS1* DKO mice were similar. **e** CFU-MK analysis generated from 150,000 cells of total marrow cells of WT and *CTTN/HS1* DKO mice plated in triplicate. **f** *CTTN/HS1* knockout did not affect MK polyploidization in vitro. **g** Platelet count and size were unaffected by the loss of CTTN and HS1. **h**, **i** Normal PPF in the WT and *CTTN/HS1* DKO mice. Results are representative of three independent experiments (*n* = 3). **h** The % of PPT bearing cells was similar between WT and *CTTN/HS1* knockout mice. **i** MKs from both genotypes displayed normal cytoplasmic extensions. **j** Platelet recovery from immune thrombocytopenia was normal in *CTTN/HS1* knockouts compared to WT. Error bars in **b**, **e**, **h** are SEM, error bars in **d**, **f**, **g**, **j** are SD

To further understand the basis of HDAC6 differential regulation of thrombopoiesis in humans and the mouse, we investigated the role of Cttn during murine PPF. Our results first showed that *hdac6* knockout MKs display significantly increased Cttn acetylation (Fig. 9a, b). To understand the role of Cttn on murine megakaryopoiesis, we used the double *Cttn/HS1*-MK-specific knockout mice (DKO); hematopoietic lineage cell-specific protein-1 (HS1) being a Cttn homolog. Western blot analysis confirmed the complete loss of both Cttn and HS1 in the knockout mice (Fig. 9c). DKO mice displayed normal femur MK number (Fig. 9d), CFU-MK growth (Fig. 9e), mean ploidy (Fig. 9f), and normal platelet count and volume compared to WT (Fig. 9g) and similar to single *HS1* ^*–/–*^ mice^32^. In vitro, PPF rate was similar in DKO mice compared to WT (Fig. 9h) with normal cytoplasmic extensions (Fig. 9i) and platelet recovery after immune depletion was normal (Fig. 9j).

## Discussion

**Fig. 10:**
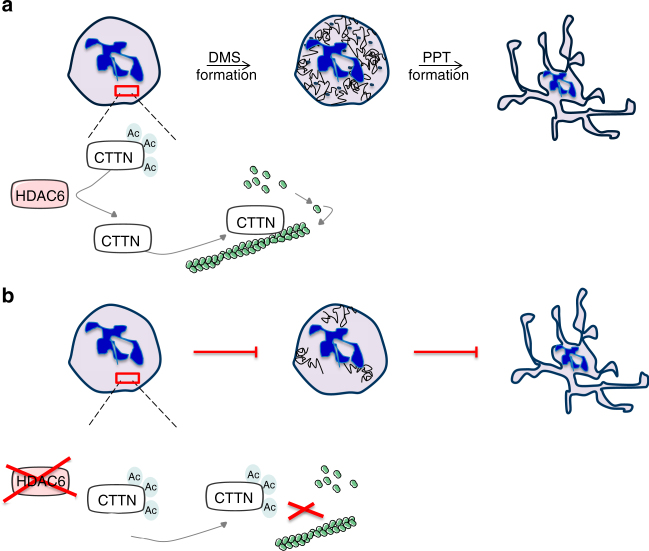
Model of HDAC6/cortactin pathway during human proplatelet formation. **a** CTTN deacetylation by HDAC6 induces filamentous actin assembly to support human platelet production. **b** HDAC6 silencing leads to CTTN hyperacetylation, decreases in its ability to bind F-actin, and inhibits its assembly impairing MK terminal differentiation

In this study, we show that the HDAC6CTTN axis plays an important role in humans in contrast to murine megakaryopoiesis (Fig. 10). Increasing evidence has revealed the role of HDAC in the hematopoietic system. Through the deacetylation of histones and a variety of nonhistone proteins, these enzymes regulate megakaryopoiesis and erythropoiesis. Class I HDACs act as catalytic subunits of multiprotein corepressors that repress gene expression by major erythro-MK transcription factors. Indeed, the HDAC1/2-containing corepressor NuRD regulates GATA1 transcriptional activity^33^^,^^34^ and mice carrying mutations disrupting the NuRD-FOG1/GATA1 interaction display a profound thrombocytopenia similar to *hdac1/2* DKO^16^. Moreover, we and others have previously shown that pan-HDACi impairs not only early human megakaryopoiesis through increased DNA double-strand breaks and p53-dependent apoptosis, but also late stages of megakaryopoiesis by another mechanism, which is mainly mediated by cytoskeleton changes in MKs that are rather due to a p53-independent mechanism^14^. It was shown in MK cell lines that pan-HDACi induces altered expression of Rho-GTPases, leading to an increase in pMLC2^35^ and also suggested that HDAC inhibition may impair MK maturation and PPF through tubulin hyperacetylation and disorganization. Thus, it is possible that the effects of pan-HDAC inhibition are due to histone hyperacetylation for early stages and due to nonhistone hyperacetylation for late stages of megakaryopoiesis^15,35^.

HDAC6 plays a central role in the regulation of the MT and actin cytoskeleton^36^. Surprisingly, it has been shown that *hdac6* knockout mice are viable without hemorrhage^24^, although the platelet count has not been reported. To gain further insights into the role of HDAC6 during human MK differentiation, we studied its expression and found that, similar to class I HDACs^37^, HDAC6 expression is barely detectable in CD34^+^ cells. HDAC6 expression strongly increases during human megakaryopoiesis. We then demonstrate that HDAC6 regulates human terminal MK differentiation without significantly affecting early stages of differentiation.

MK differentiation is regulated by several transcription factors and interestingly, we found a direct binding of RUNX1 and FLI1 to *HDAC6* promoter close to the TSS region. Confirming our result, *RUNX1* knockdown in human MK leads to a decrease of *HDAC6* expression. Our results are in accordance to a reported genome-wide analysis of simultaneous binding sites of RUNX1 and FLI1 transcription factors in primary human MKs^38^. These available data also revealed that GATA1, SCL, and at a lesser extent GATA2 bind to HDAC6 promoter. Altogether, these results suggest that human HDAC6 may be regulated at a different extent by these MK transcription factors.

Specific inhibition of HDAC6 with Tubastatin A, ACY1215, and two different shRNAs induced a strong decrease in PPF. Taking into account previous published results, we first thought that HDAC6i-induced MT hyperacetylation was responsible for the inhibition of PPF. Indeed, HDAC6 inhibition resulted in a strong increase in ac-tubulin, suggesting that HDAC6 is the main TDAC in MKs. Furthermore, Sirtuin2, a class III HDAC^29^ did not play an important role in MK ac-tubulin because its inhibition on top of HDAC6 inhibition did not further increase tubulin acetylation. Strikingly, this induction of tubulin hyperacetylation did not render the MT cytoskeleton more resistant to nocodazole. The role of MT acetylation on its dynamics has long been a matter of controversy. Initially, numerous studies revealed that HDAC6i-induced tubulin hyperacetylation enhances MTs stability^23,39,40^. This was challenged by studies showing that the highly acetylated MTs of NIH3T3 cells did not induce a resistance to nocodazole^41^. These differences may be cell-type dependent. Moreover, we found that tubulin 40KR, a non-ac-tubulin mimic, failed to reversed the PPF defect induced by HDAC6i, suggesting that tubulin acetylation is not involved in the regulation of PPF. Our results do not support the findings that implicate MT hyperacetylation as the mechanism of HDACi-induced PPF defect^15^. In this study, it was not shown that the defect in PPF induced by the pan-HDACi LBH589 was due to tubulin hyperacetylation in MKs as only a cytoskeleton disorganization was observed. Surprisingly, our study revealed that *HDAC6* knockdown in human MKs led to a disturbed MK maturation, leading to a heterogeneous defect in DMS development associated to an unusual large MVB and a defect in granule biogenesis assessed by the enlarged α-granules. As HDAC6 expression is regulated by MK-restricted transcription factors, we can hypothesize that the thrombocytopenia associated to the loss-of-function mutations in *RUNX1*, *FLI1*, and *GATA1* in human pathologies may be partly explained by a decreased expression of HDAC6, more particularly the abnormalities in DMS and granule development. We thus oriented our investigations toward the F-actin-binding protein CTTN, another major HDAC6 target^22,39,42^ expressed in mature MKs and its expression increased in a megakaryoblastic cell line after PMA treatment^43^. Herein, we showed that CTTN expression greatly increased during human megakaryopoiesis and that it colocalized with the actin cytoskeleton. We showed for the first time that CTTN depletion strongly decreased the percentage of MKs forming PPTs in vitro. As expected, both HDAC6i and shRNA induced a dose-dependent increase in CTTN acetylation, leading to a decrease in the F-actin content and a reduction in its capacity to bind F-actin, hence impairing actin polymerization and branching. A CTTN mutant that mimics its deacetylated form (CTTN-9KR) efficiently counteracted HDAC6i-induced actin depolymerization and PPF decrease.

The main role of HDAC6 on MK terminal differentiation seems to be related to the organization of actin cytoskeleton and its role on PPF is controversial. It is well demonstrated that the driving forces of PPF are mediated by tubulin but not by actin. However, actin polymerization plays a central role in regulating DMS formation^9^ and PPT branching^11^. Any defect in DMS organization is associated with a marked defect in PPF. The link between CTTN and granule development remains to be investigated. However, recently, it was shown that in humans, a germline mutation in *SRC* that has CTTN as the target led to a thrombocytopenia with a major defect in alpha-granules^44^. ARP2/3 complex is involved in DMS organization. It has been shown that numerous cellular assembly processes require activation of ARP2/3 by several nucleation-promoting factors such as CTTN and N-WASP^45^. We recently demonstrated that human N-WASP is also critical for PPF^46^ ; thus, we can hypothesize that the synergy between HDAC6-CTTN and N-WASP led to a disturbed MK maturation leading to a heterogeneous defect in DMS development associated to a defect in granule biogenesis. Currently, the role of N-WASP in murine megakaryopoiesis is unknown. Thus, it is possible that in the mouse, the regulation of the actin cytoskeleton in late MK differentiation is more dependent on PAK2 and WAVE^9,47,48^ than on CTNN and N-WASP. However, we cannot exclude that CTTN may act through other partners such as dynamin 2 whose knockout leads to a defect in DMS formation and PPF^49^. However, this hypothesis seems less likely as the results on dynamin 2 were obtained in the mice where we show that CTTN is not involved.

The precise mechanism by which mouse MKs compensate for the lack of Hdac6 and Cttn remains partially unresolved. A quantitative proteomic study, monitoring the protein level of other HDAC family members such as Hdac3–7, Hdac9, and Hdac11 and Sirt2, showed that Hdac6 deficiency does not alter their expression in the mouse liver^50^. Herein, as others, we also reported that *Hdac6* inactivation in mice does not affect the acetylation level of histone proteins, showing the absence of a compensatory increase in the HDAC activity^24^. Similarly, mice lacking Cttn and its hematopoietic analog HS1 undergo normal terminal megakaryopoiesis and are dispensable for MK podosome formation and platelet function^51^. One hypothesis would be that other actin organizers such as WAVE and Arp2/3 complex may be responsible for the lack of a phenotype in the *cttn*/*HS1* double-knockout mice.

Taken together, this result and others observed in vitro and in vivo for DIAPH1^52,53^ and WASP^54^ confirms that the regulation of the cytoskeleton in human and murine MKs is different.

Our in vitro studies have been confirmed in vivo as recently, the use of ACY1215 (ricolinostat) in human monotherapy or in combination with other therapy has been shown to lead to thrombocytopenia^55^. From our data, it is expected that HDAC6 inhibition will lead to a milder thrombocytopenia than with pan-HDACi because HDAC6 inhibition will alter only late stages of megakaryopoiesis. In future clinical trials, it will be important to determine if the thrombocytopenia is related to the specific HDAC6 inhibition or to an off-target effect of the inhibitor on other HDAC. These findings open new avenues in the understanding of the role of different HDAC and CTTN during human megakaryopoiesis and may suggest that this axis is implicated in pathology and in the thrombocytopenia associated with HDACi in cancer therapy.

## Methods

### In vitro culture of MKs derived from human CD34^+^ cells

CD34^+^ cells were obtained, in agreement with our institutional ethics committee (Assistance Publique des Hôpitaux de Paris) and in accordance with the Declaration of Helsinki, from leukapheresis samples. CD34^+^ cells were isolated by a positive selection using immunomagnetic bead cell-sorting system (AutoMacs; Miltenyi Biotec) and cultured in serum-free medium in the presence of recombinant human thrombopoietin (rhTPO; 10 ng/mL; Kirin Brewery). Two HDAC6-selective inhibitors, Tubastatin A (Sigma) and ACY1215, generously provided by Simon Jones (Acetylon Pharmaceuticals Inc.), were used.

### Mice

*hdac6* ^*–/–*^ mice^24^ were housed at the Gustave Roussy Animal Facilities (Villejuif, France) and *HS1* ^*–/–*^ */CTTN* ^*fl/fl*^ *Pf4*.*Cre* at the Institute of Cardiovascular Sciences. All experiments were carried out according to national and institutional guidelines.

### Immune thrombocytopenia

To induce thrombocytopenia, anti-CD41 was injected into *hdac6* knockout mice, CTTN, and their WT littermates. Platelet count from both genotypes was determined at 24, 48, and 72 h for *hdac6* KO mice and from 24–168 h for *cttn* KO mice.

### DNA manipulation, shRNA cloning, and lentivirus production

shRNA constructs targeting the human HDAC6, αTAT, and CTTN (primers) sequences were cloned, as previously described, in the pRRL-pGK-GFP lentiviral vector (Open Biosystems).

40KR-Tubulin and 9KR-CTTN were kindly supplied, respectively, by F. Saudou (Institut Curie, Orsay, France) and Dr. Zhang (Tampa, FL, USA). Human CTTN plasmid was purchased from Addgene and CTTN silencing was carried out using the NEB-directed mutagenesis kit. The different constructs were cloned into the pRRL-EF1α-MCS-PGK-mCherry lentiviral vector. NES-mutant HDAC6 was generated using the Q5 Site-Directed Mutagenesis Kit according to the manufacturer’s instructions. Lentiviruses were produced and delivered into the cells^4^. pRRL lentiviruses were produced by 293-T cells cotransfected by pCMV-Gag-Pol and pCMV-VSV-G plasmids (Open Biosystems). Supernatants were collected at 48, 72, and 96 h after transfection, and were pooled and concentrated by ultracentrifugation. Virus stocks were kept frozen at –80 °C.

### Cell transduction

CD34^+^ cells were cultured for 5 days in serum-free medium in the presence of TPO (10 ng/mL) and SCF (50 ng/mL) and transduced with lentiviral particles for 3 h followed by a second transduction. Cells were sorted 48 h after transduction on the expression of CD41, GFP, and/or Cherry by an Influx cytometer (Becton Dickinson).

### Measurement of ploidy

Hoechst 33342 (10 μg/mL; Sigma) was added at day 10 in human MK cultures for 1 h 30 min at 37 °C. Cells were then stained with directly coupled MoAbs: anti-CD41 allophycocyanin (APC) and anti-CD42 phycoerythrin (1:100; BD Biosciences) for 30 min at 4 °C. The ploidy was measured in the CD41^+^/CD42^+^ cell population by a LSRII flow cytometer equipped with three lasers (360, 480, and 560 nM excitation). The mean ploidy of MKs was measured^4^. The total BM cells from WT mice or *hdac6* KO littermate were purified and co-stained with FITC-conjugated anti-CD41 antibody and propidium iodide in a hypotonic citrate solution containing 50 μg/mL RNase. The mean ploidy of MKs was measured^4^.

### Real-time quantitative RT-PCR

The total RNA was extracted with Trizol (Sigma) and reverse transcription was performed using 500 ng of total RNA with SuperScript II Reverse Transcriptase protocol (Life Technologies). Quantitative PCR was performed using an Applied Biosystems 7500 Real-Time PCR System. Amplification reactions were carried out using Power SyberGreen Master Mix (Life Technologies) according to the manufacturer’s recommendations. The expression levels of all studied genes (*hdac6*, *hdac1*, *hdac2*, *hdac3*, *CTTN*, *αTAT*, *sirt1*, and *sirt2*) were calculated relatively to the expression of HPRT and PPIA as endogenous housekeeping controls. Primer sequences are listed in Supplementary Table 1.

### Quantification of MK progenitors in semisolid cultures

CD34^+^ cells were cultured in serum-free fibrin clot assay with 10 ng/mL of TPO, 50 ng/mL of SCF (kindly respectively provided by Kirin, Tokyo, Japan and Biovitrum AB, Stockholm, Sweden) and 10 ng/mL of IL6 (Miletnyi Biotech, France), and in the presence or absence of various concentrations of Tubastatin A and ACY1215 ranging from 0.05 to 5 µM. After 12–14 days of incubation at 37 °C, MK colonies were revealed and enumerated^14^.

A total of 150,000 cells from the total bone morrow of WT and *hdac6* KO mice cells were seeded in triplicate and allowed to form CFU-MK for 5 days. MK colonies were revealed by acetylcholinesterase staining^14^.

### Effect of HDAC6 on cellular growth and CD41^+^42^+^ generation

To assess cellular proliferation, 50,000 CD34^+^ cells were seeded in the presence of TPO ± Tubastatin A or ACY1215, and viable cells were counted using trypan blue exclusion at day 6 of differentiation. The quantification of the total number of CD41^+^ CD42^+^ cells generated at day 6 of culture in the presence or absence of increasing doses of HDAC6 inhibitors was derived from the total cell number and the % of CD41^+^ CD42^+^ cells analyzed by flow cytometry.

### PPF assay and determination of platelets produced in culture

CD41^+^-sorted cells were seeded in triplicate at day 8 of culture in a 96-well plate (3000 cells/well) in serum-free liquid medium supplemented with 10 ng/mL rhTPO with or without increasing the concentration of Tubastatin A and ACY1215. MKs displaying PPTs were counted^14,52^. Quantification of platelets produced in vitro was assessed by flow cytometry^56^. Briefly, platelet events were collected without gating using a log scale for FSC and SSC. An analytical gate was determined based on scatter properties of normal blood platelets treated similarly. This gate excluded large contaminating cells (MKs) and a small debris or microparticles. Culture-derived platelets were enumerated as CD41 events with the same scatter properties as blood platelets.

### Cell cycle analysis

CD34^+^ cells were grown for 48 h in serum-free liquid medium with rhTPO (10 ng/mL) in the presence or absence of HDAC6-specific inhibitors. Cells were stained for 30 min at 4 °C with propidium iodide (50 µg/mL; Sigma) in a hypotonic citrate solution containing 50 µg/mL RNase (Sigma). The number of cells in phase G0, S, and G2/M was analyzed by Canto II flow cytometer.

### Assessment of cell viability, apoptosis, and maturation

CD34^+^ cells were seeded in triplicate in the presence or absence of increasing doses of Tubastatin A and ACY1215. Cell viability was measured by trypan blue exclusion, and cell proliferation was quantified by manual cell counting. Apoptosis was investigated by AnnexinV PE and DAPI flow cytometry analysis.

### Immunofluorescence and confocal imaging

Immunofluorescence was performed on CD41^+^ cells cultured with or without inhibitors^4^. In brief, cells were allowed to adhere on poly-l-lysine-coated slides for 1 h at 37 °C, fixed in 4% PFA, permeabilized with 0.2% of Triton, and blocked with 1% BSA before antibody labeling. Cells were examined under a Leica-SpE confocal microscopy with ×63/1.4NA oil objective. The following antibodies were used: mouse anti-ac-tubulin (1:300, Sigma T7451), mouse anti-Tubulin (1:100, Sigma T5293), rabbit anti-HDAC6 (1:100, Cell Signaling 7558), and rabbit anti-Cortactin (1:100, Millipore 05-180). FITC-conjugated phalloidin (1:500, Sigma P5282) was used to stain actin cytoskeleton, mouse anti-CD63 (1:100, Sigma SAB4700215, clone MEM 259), and rabbit anti-VWF (1:1000, Dako A0082). Appropriate secondary antibodies conjugated with Alexa 488, Alexa 546, or Alexa 633 (Molecular Probes) were used. DAPI (Molecular Probes) was applied for nucleus staining. Three-dimensional image analyses were performed with Zeiss Image Examiner software.

### Western blot analysis

CD41^+^ cells were harvested at different times of culture, washed once in 1× PBS, and lysed in Nonidet P40 (NP40) buffer containing protease and phosphatase inhibitors (Roche)^14^. Lysates were gently sonicated on ice and protein concentration was assessed using Bradford assay (Bio-Rad). Protein samples were subjected to SDS–polyacrylamide gel electroporesis. After transfer, nitrocellulose membranes were blotted with the following 1/1000-diluted antibodies: anti-HDAC6, anti-acetylated cortactin, and anti-cortactin (from Millipore, catalog number 07-732, 09-881, and AB3887), anti-ac-tubulin, anti-tubulin, and anti-actin (Sigma, catalog number T7451, T9026 clone DM1A, and A2228 clone AC-74), anti-acetylated histone H3 (Cell Signaling, catalog number 4353), and anti-HSC70 (Abcam, ab19136), followed by revelation by horseradish peroxidase-linked secondary antibodies and Chemiluminescent HRP Substrate (Millipore) using IQuant Instrument (GE Healthcare).

### G/F actin measurement

CD41^+^ cells were sorted on day 8 and treated with 1 µM of either Tubastatin A or ACY1215 for 48 h. Cells were then harvested and washed twice with ice-cold PBS and resuspended in lysis buffer containing 50 mM PIPES, 50 nM Nacl, 5 nM MgCl_2_, 5 mM EGTA, 5% glycerol, 0.1% NP40, Triton and Tween 20, and protease inhibitors and centrifuged for 1 h at 100,000×*g* at 37°C. Supernatants containing G-actin were removed and pellets (F-actin) were resuspended in an equal lysis buffer containing 1 µM cytochalasin D and incubated for 1 h on ice to stabilize actin filaments. Equal volumes were loaded and subjected to western blot analysis.

### Migration assessment

CD34^+^ cells were cultured in serum-free medium in the presence of rhTPO and SCF with or without HDAC6i. At day 5 of culture, 100,000 cells were allowed to migrate through an 8-µm transwell (Millipore) under SDF-1 (100 ng) for 3 h at 37 °C. Cells were then harvested, stained with specific antibodies against CD34, CD41, and CD42, and analyzed by flow cytometry.

### Tubulin depolymerization assay

HDAC6i-treated and untreated CD41^+^ MKs were plated on collagen-coated slides for 1 h, and then incubated with serum-free medium containing 1 μM nocodazole (Sigma, France) for the indicated times to enhance MT depolymerization. Cells were carefully washed, fixed with 4% PFA for 10 min at 37 °C, and permeabilized for 10 min with 0.2% Triton as reported above. Cells were then stained with an antibody against tubulin (Sigma) and imaged by Leica confocal microscope.

### Chromatin immunoprecipitation

ChIP experiments were performed with ChIP-IT according to the manufacturer’s instructions (active motif). Briefly, cells were cross-linked with 37% paraformaldehyde and the reaction was stopped with glycine, washed twice in cold 1× PBS, lysed in SDS lysis buffer completed with protease inhibitor cocktail (Millipore), and sonicated using Covaris S220 System. Antibodies of 5 µg (anti-rabbit IgG, ab171870, Abcam, Paris, France; anti-RUNX1, ab23980, Abcam, Paris, France; and anti-FLI1, ab15289, Abcam, Strasbourg, France) were added and incubated overnight. Immunoprecipitated chromatin was eluted from the magnetic beads after several washes and the cross-links of these sequentially immunoprecipitated protein–DNA complexes were then reversed, and the DNA was analyzed by real-time PCR (Applied Biosystems) for HDAC6 promoter using HDAC6prom-F1: ATGAGGAAACGGAGCACAGAA and HDAC6prom-R1: CGGGACTTAACCATGTGACTTTG.

### Statistical analyses

Analysis was performed using Prism software (GraphPad Software Inc) and unpaired *t*-tests were used for the statistical analyses. Asterisks indicate significant differences: ^*^ *p*-value < 0.05, ^**^ *p*-value < 0.01, and ^***^ *p*-value < 0.001.

### Data availability

Data supporting the findings of this study are available within the article and its Supplementary Information files and from the corresponding author upon reasonable request.

## Electronic supplementary material

Supplementary Information
