# Supplementary material for: Critical role of the HDAC6–cortactin axis in human megakaryocyte maturation leading to a proplatelet-formation defect
Source: Nat Commun. 2017 Nov 27;8:1786. doi: 10.1038/s41467-017-01690-2 (PMC5702605; doi:10.1038/s41467-017-01690-2)
Supplement: Supplementary file 1 — Supplementary Information [file 41467_2017_1690_MOESM1_ESM.pdf]

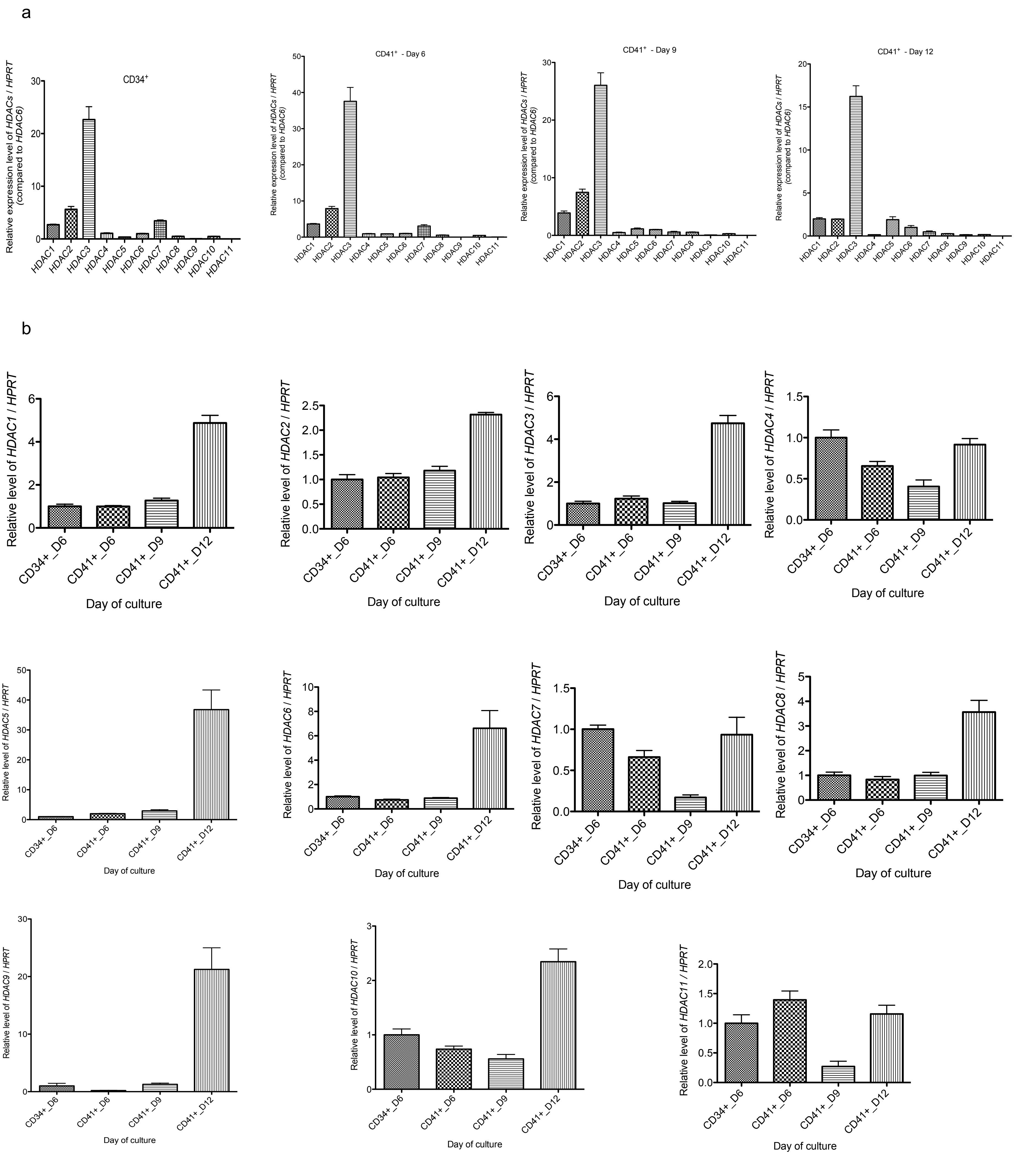

Supplementary Fig. 1: Modulation of the different HDACs expression during megakaryocyte differentiation. CD34^{+} cells were differentiated to MK and sorted on expression of CD34 and CD41 at day 6 of culture. A fraction of the CD41^{+} cells were grown for 3 and6 additional days allowing MK maturation **(a)** Relative *HDACs* mRNA level expression to *HPRT* mRNA in the CD34 and CD41 Day 6, Day 9 and day 12.**(b)**Relative *HDACs* mRNA level expression to *HPRT* mRNA . Bar graphs represent mean ± SD.

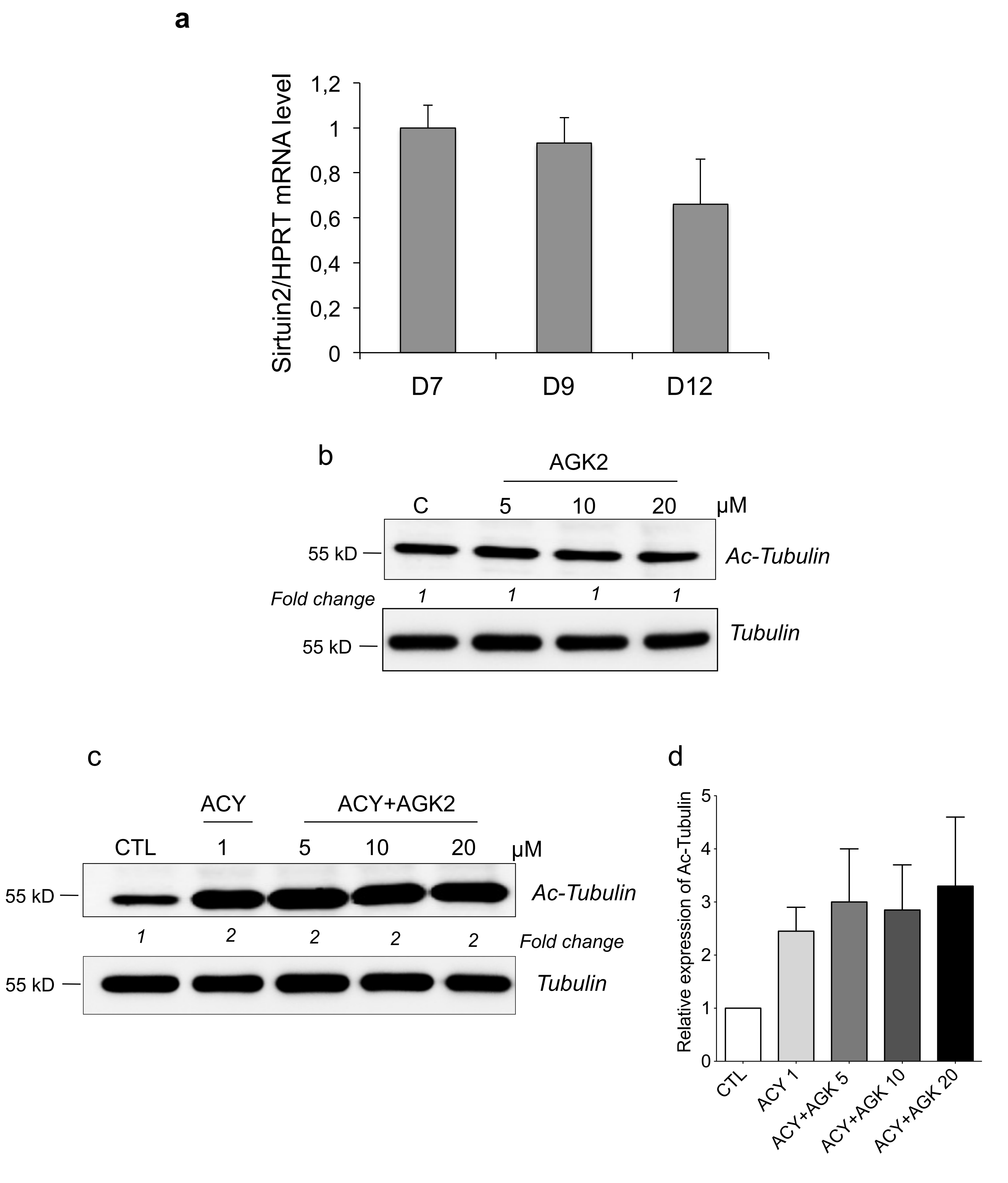

Supplementary Figure 2. HDAC6 is the main Tubulin deacetylase (TDAC) in MK. (a) Sirtuin2 expression during megakaryopoiesis. Relative *Sirtuin2* mRNA level expression to HPRT at different times of human MK maturation (Day 7, 9, and 12) Bar graph represent mean ± SD. (b-d) Sirtuin2 inhibition has no effect on tubulin acetylation in MK. $CD41^{+}$ cells were treated with increasing doses of AGK2 ranging from 5 to 20µM in absence, n=2 (B) or presence of 1µM of ACY1215 (c-d). Bar graph represent mean ± SEM

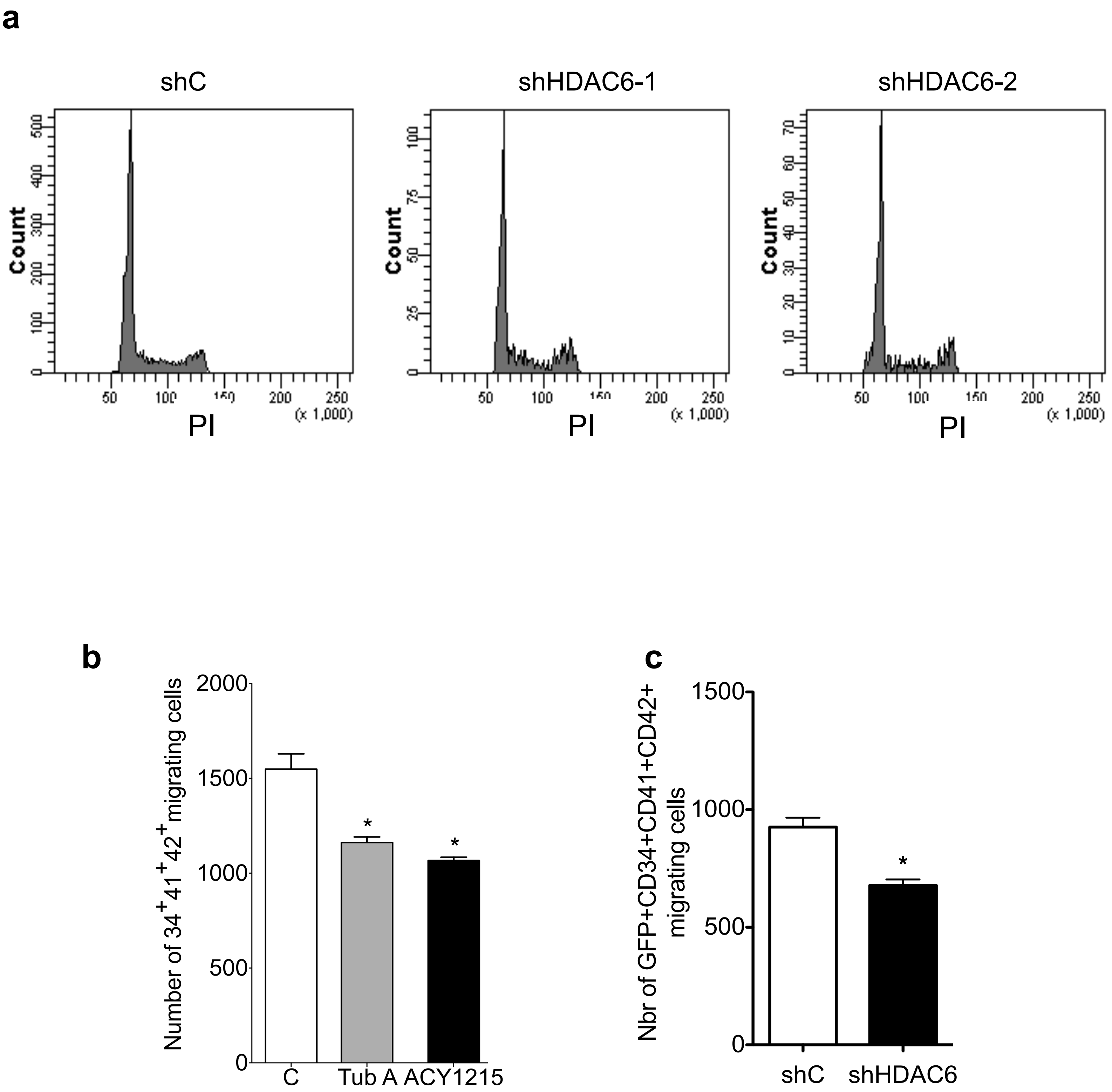

Supplementary Figure 3. HDAC6 inhibition does not impair cell cycle progression and decreases moderately MK migration. **(a)** Cell cycle analysis of $CD34^+$ cells transduced with the control lentivirus shC, shHDAC6-1 and shHDAC6-2. **(b-c)** Tubastatin, ACY1215 and shHDAC6 decrease slightly MK migration. Unpaired Student's t-test $*p = 0.0343$. Bar graphs represent mean ± SEM

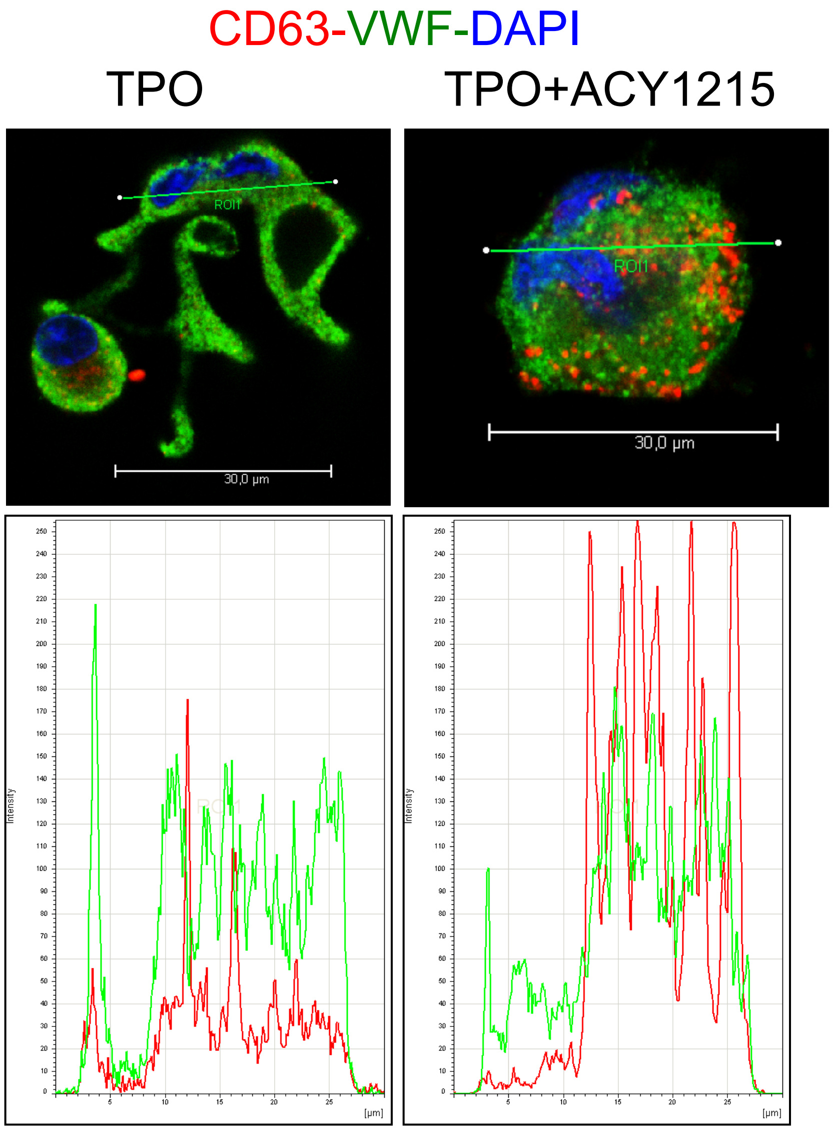

Supplementary Figure 4: Colocalization of CD63 and VWF in MK treated with 1µM of ACY1215. CD63 (red), VWF (green) Dapi (blue) compared to control MK

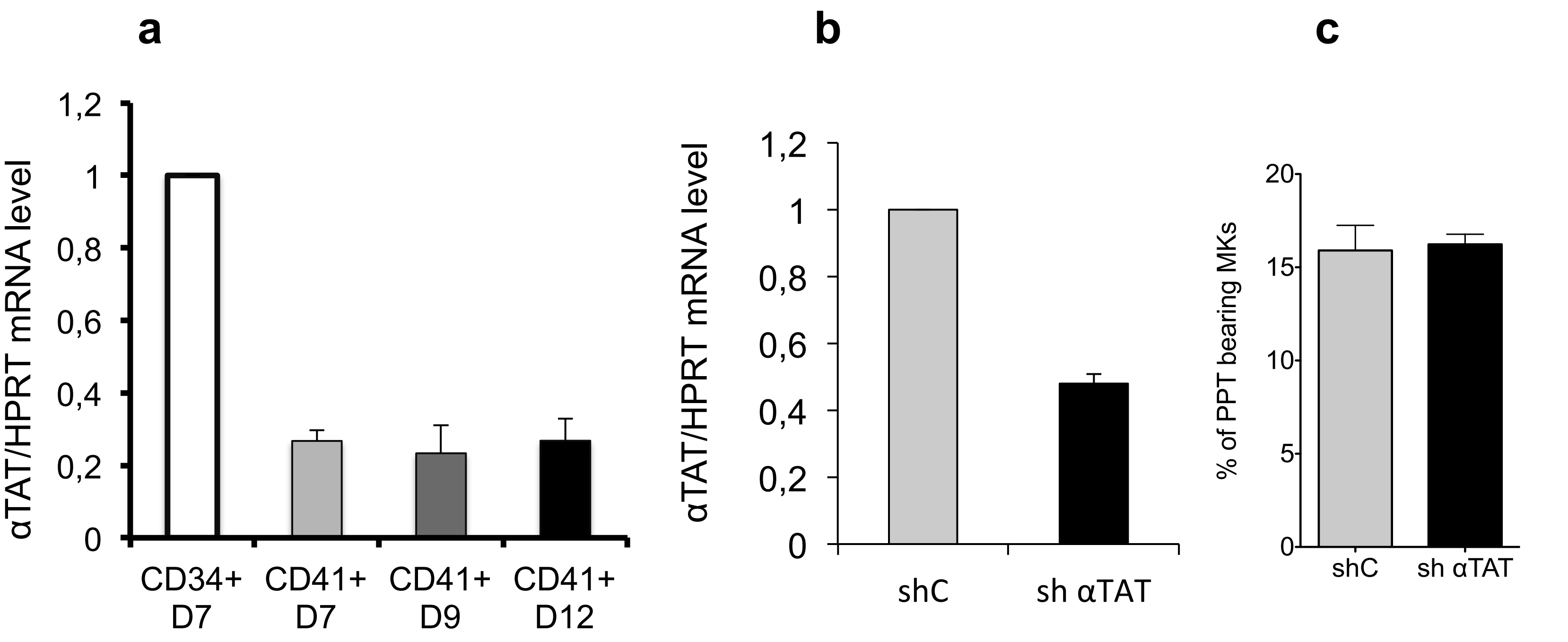

Supplementary Figure 5. αTAT silencing has no effect on PFF in vitro. (a) αTAT expression during MK maturation. (b-c**)** CD34^{+} cells were transduced with either sh Control (C) or shαTAT and sorted 48h later on GFP and CD41 expression. A fraction of sorted cells was subject to RT-qPCR analysis (b) and PPF (c) assays. Results are representative of three independent experiments. Bar graphs represent mean ± SD (a-b) and SEM (c)

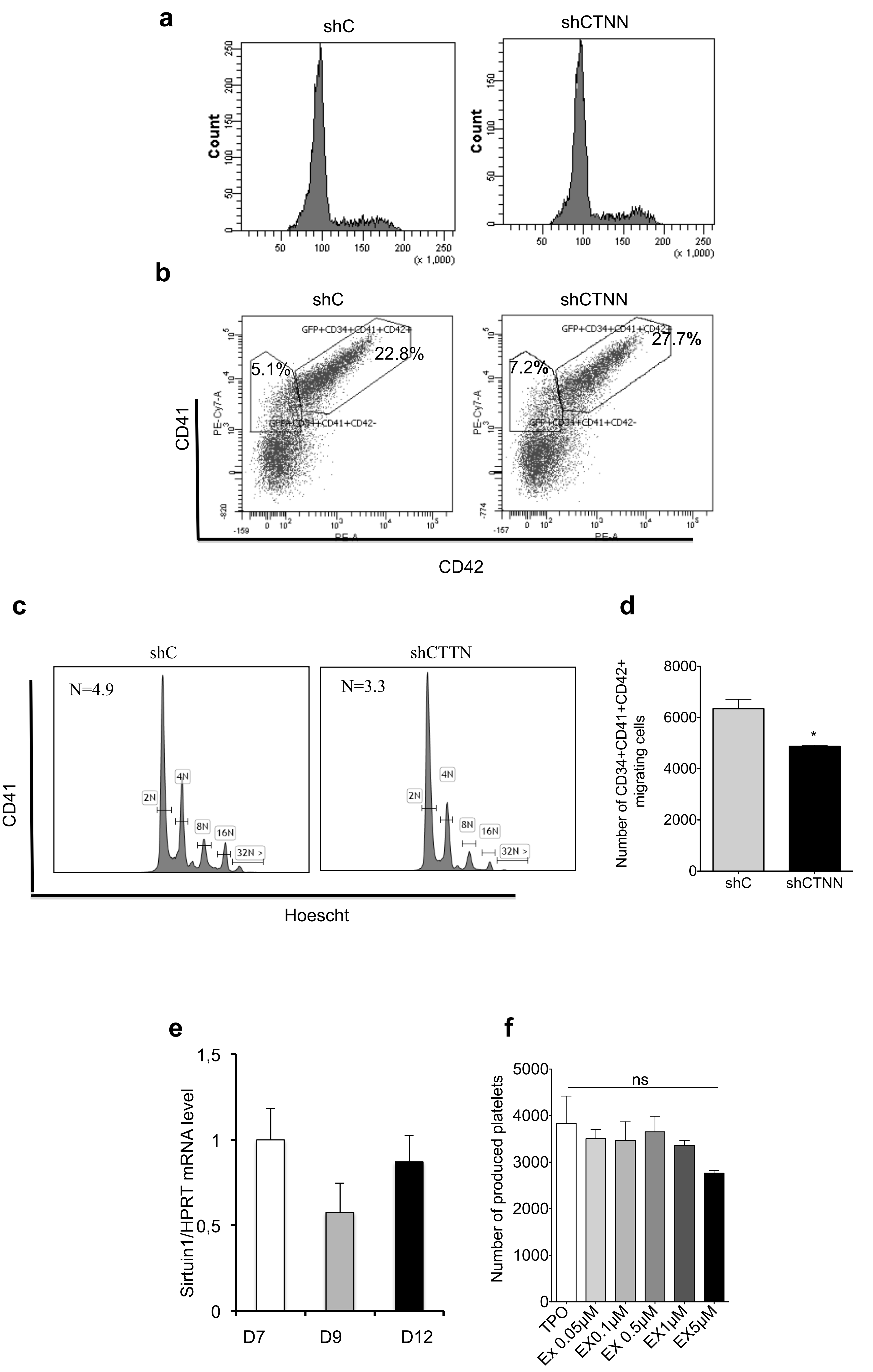

Supplementary Figure 6. ***CTTN*** **knockdown** (**a**) does not affect cell cycle progression and (**b**) MK maturation, but (**c)** decreases MK mean ploidy, shC N=4.9; shCTTN N=3.3 and (d) slightly MK migration, Unpaired Student's t-test *P<0.05. (**e**) Relative *Sirtuin*1 mRNA level expression to HPRT at different time of MK maturation (Day 7, 9, and 12). (**f**) Sirtuin1inhibition has no effect on *in vitro* platelet production evaluated by flow cytometry.

Supplementary Figure 7

Uncropped blots related to Figure 1b

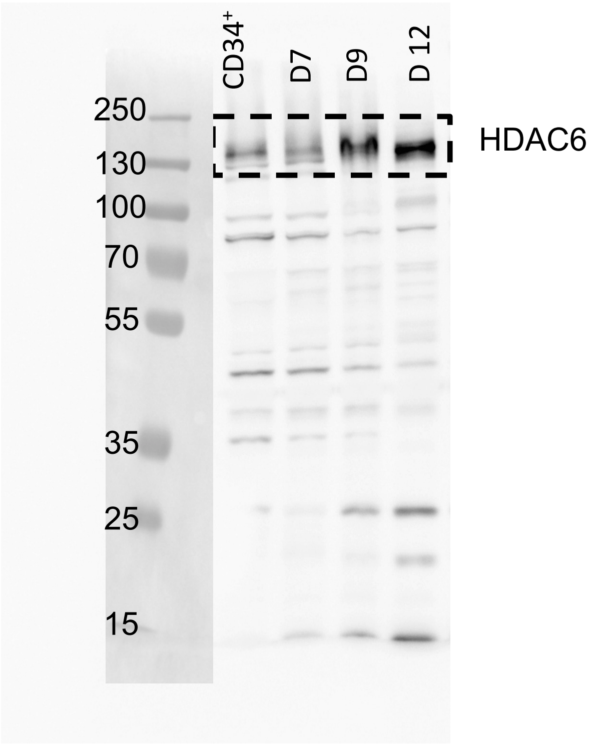

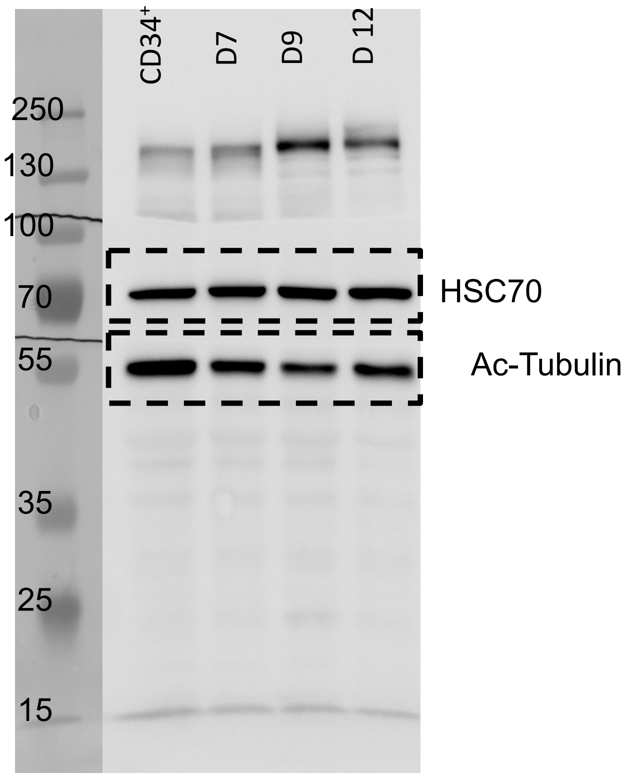

Supplementary Figure 7

Uncropped blots related to Figure 2 a and g

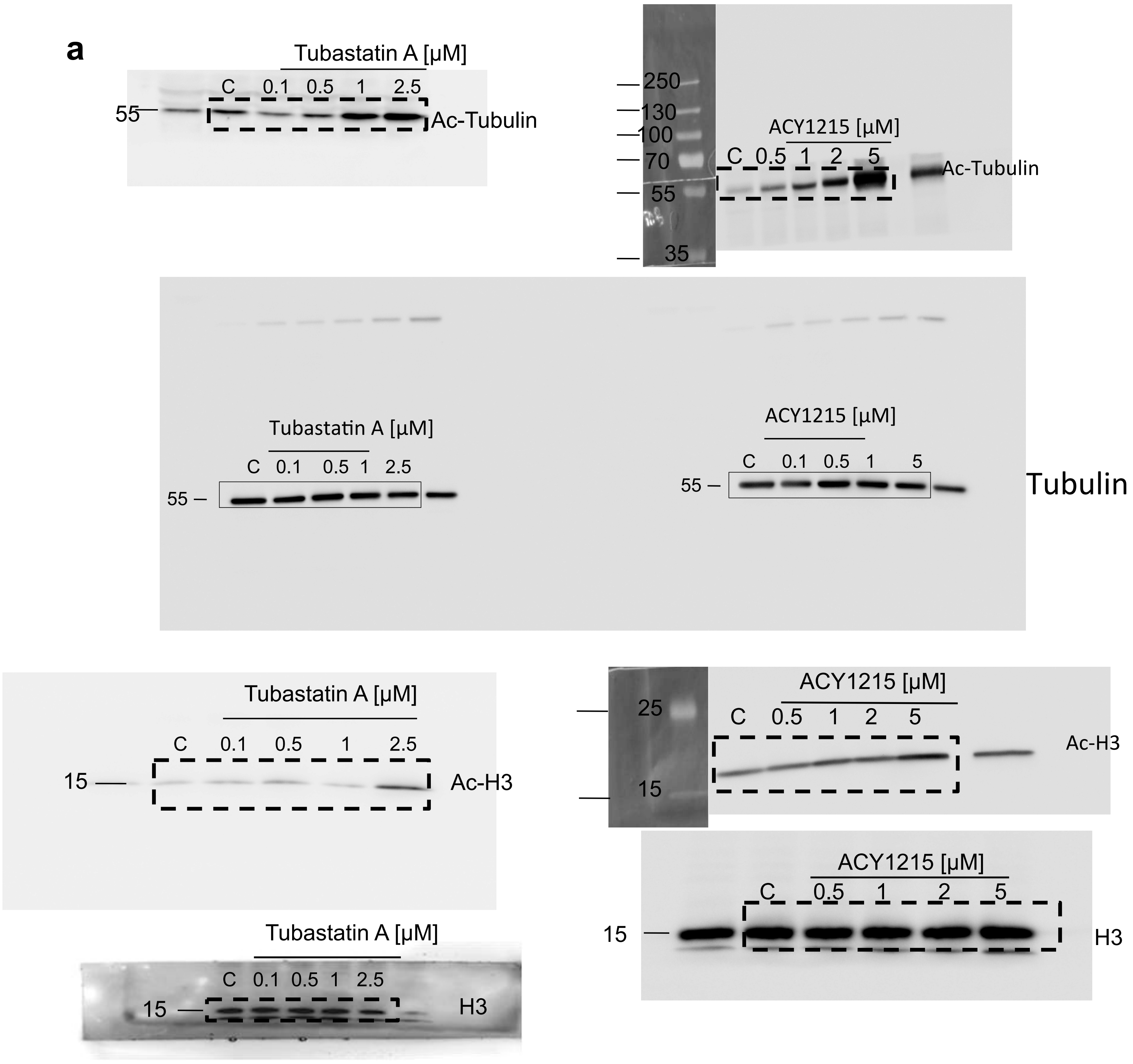

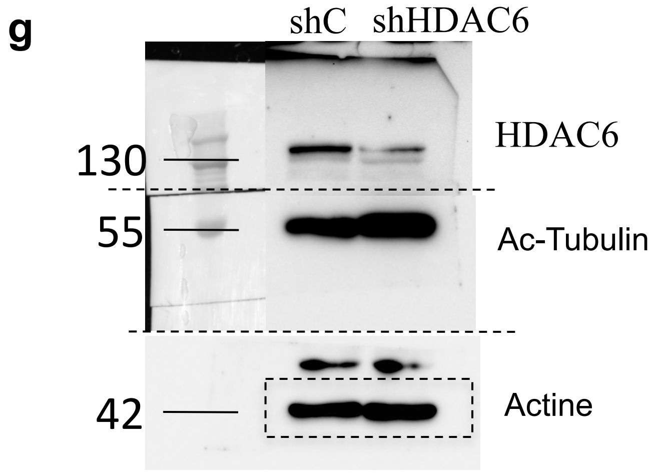

Supplementary Figure 7

Uncropped blots related to Figure 4 a and i

a

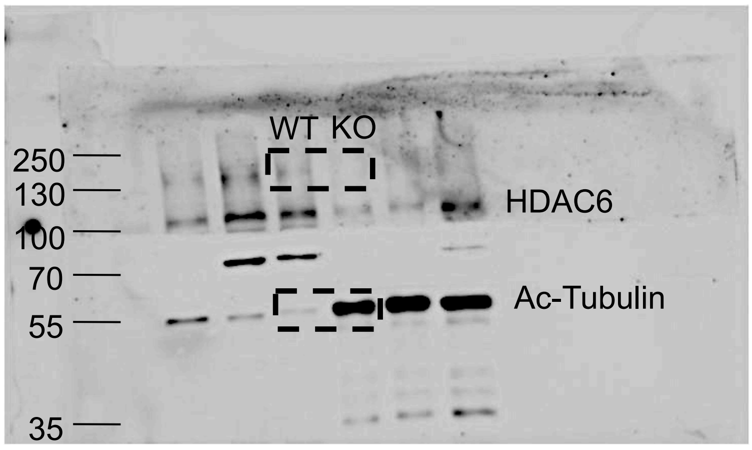

i

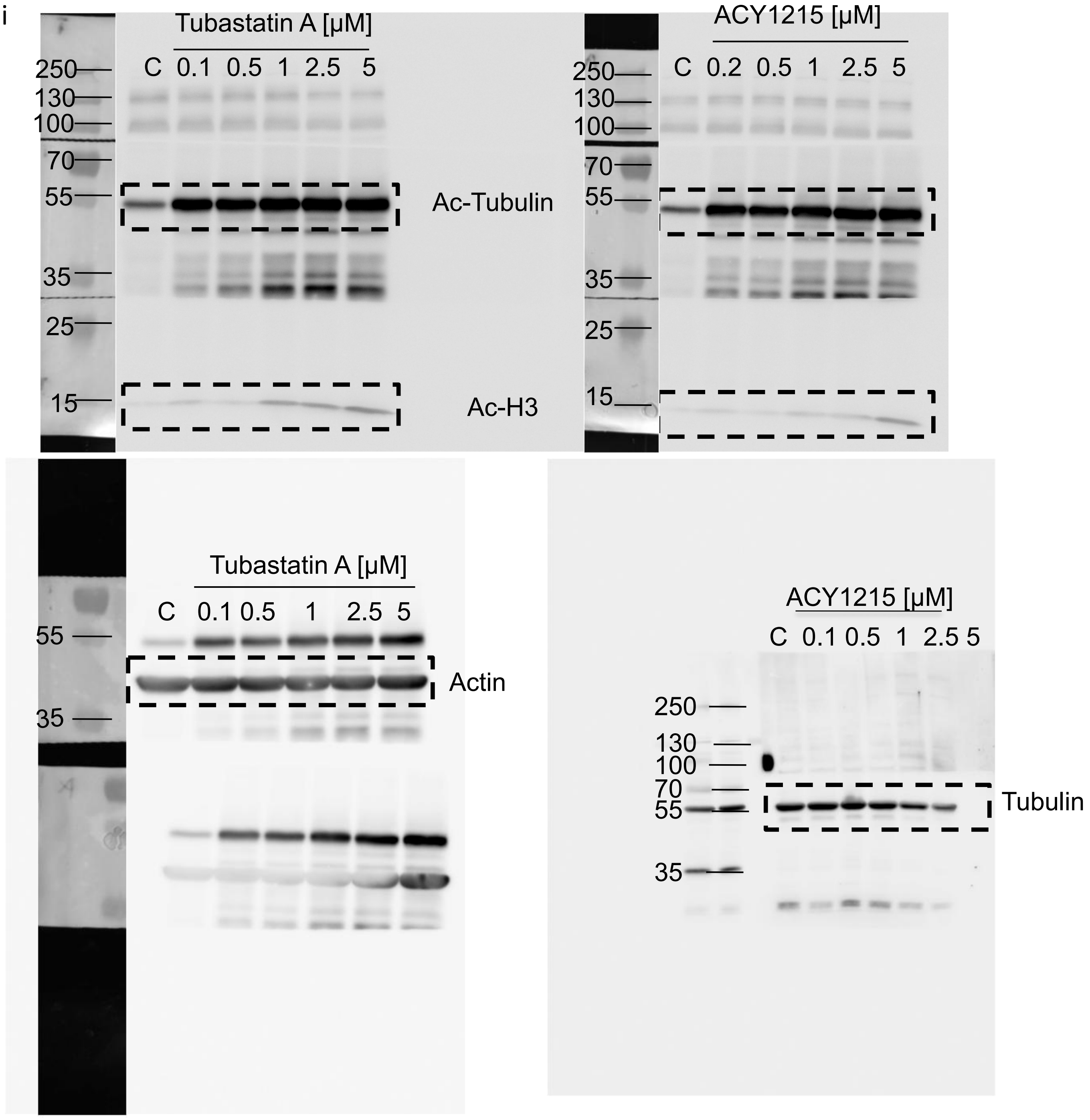

Supplementary Figure 7

Uncropped blots related to Figure 7 b and f

b

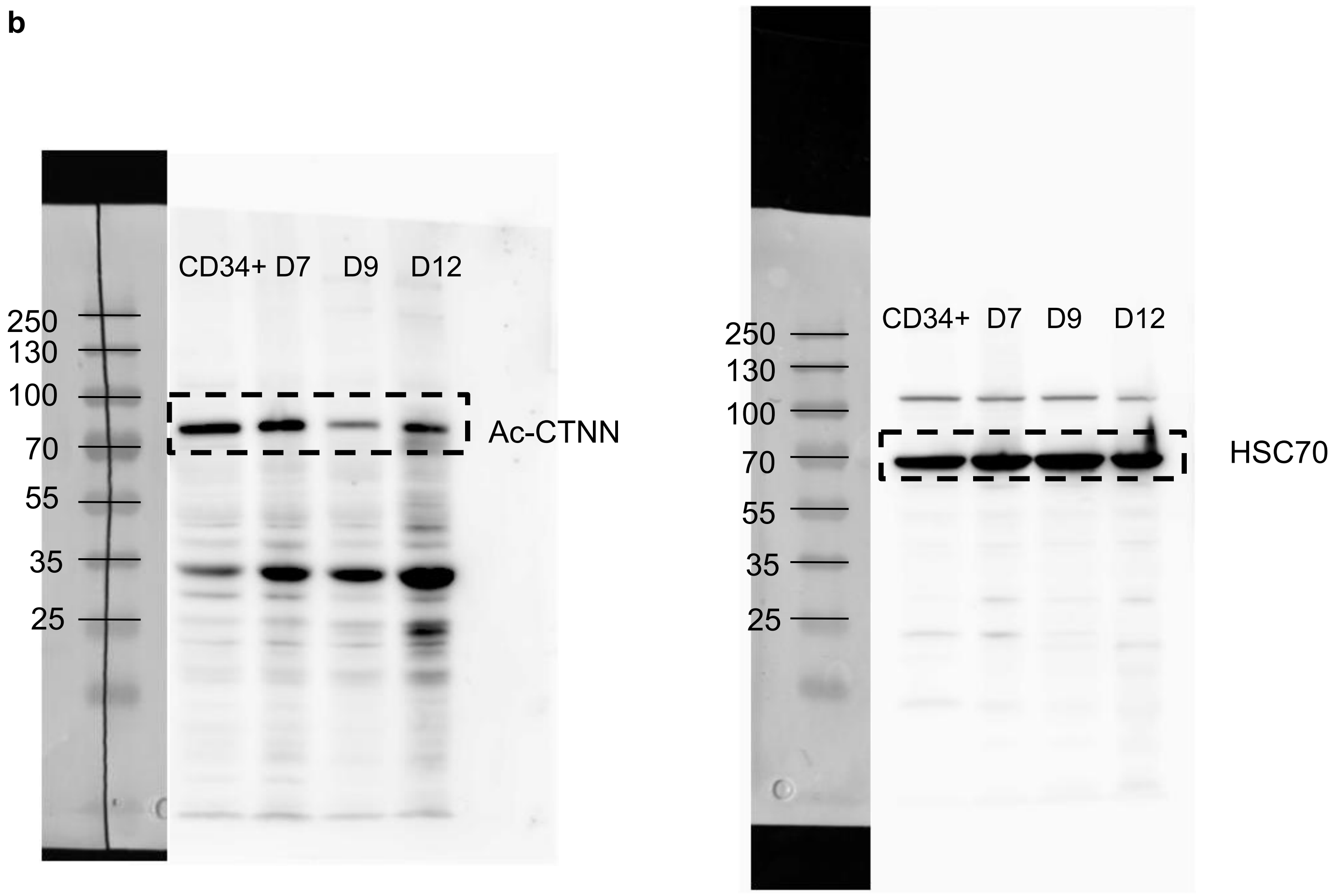

f

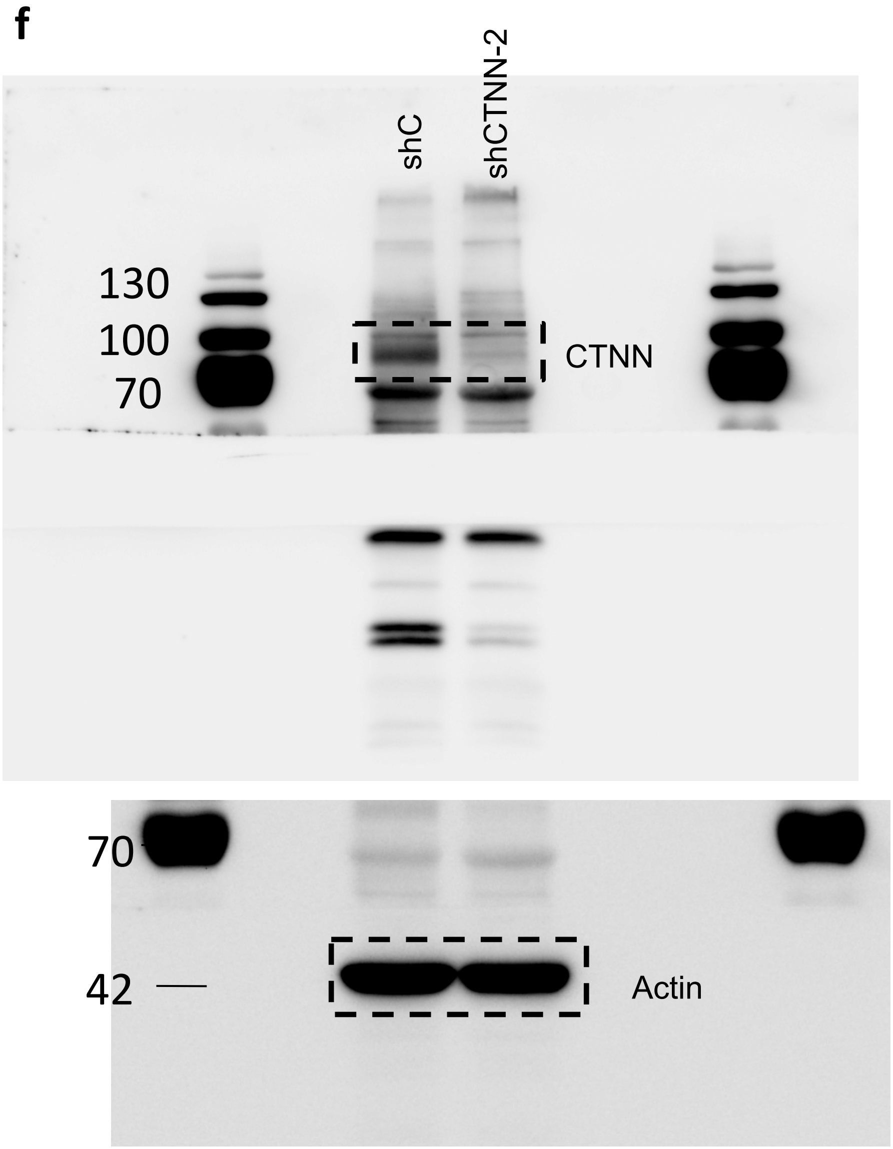

Supplementary Figure 7

Uncropped blots related to Figure 8a, 8c ,g

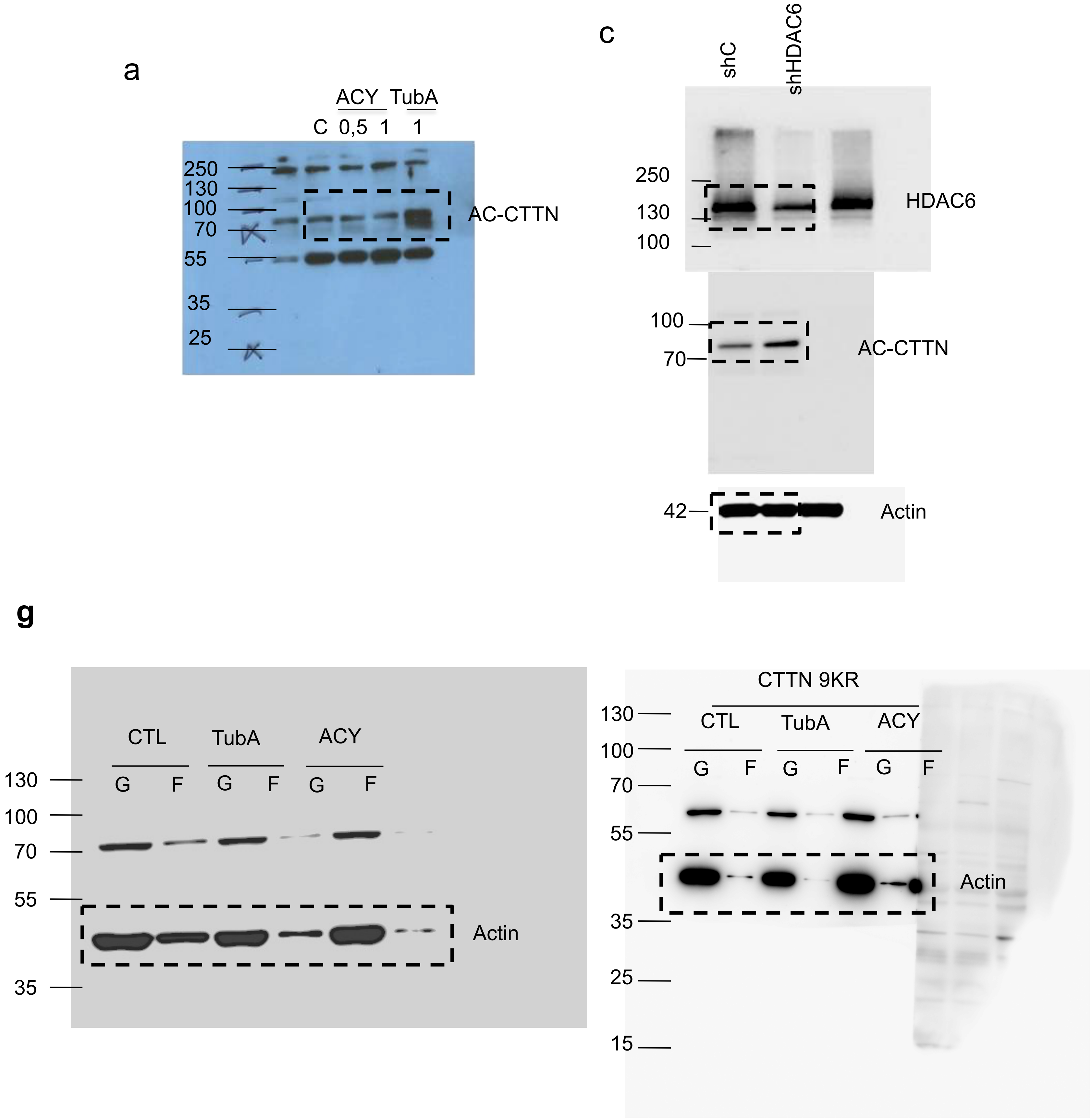

Supplementary Figure 7

Uncropped blots related to Supplementary Figure 2 c

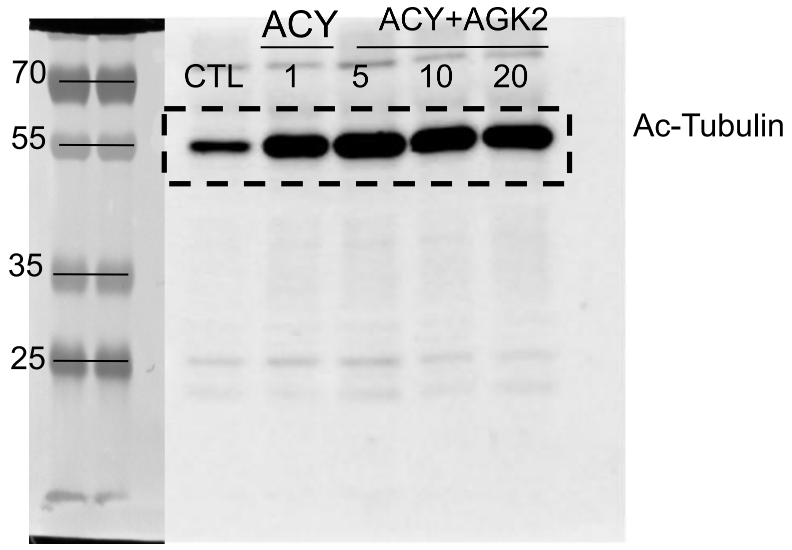

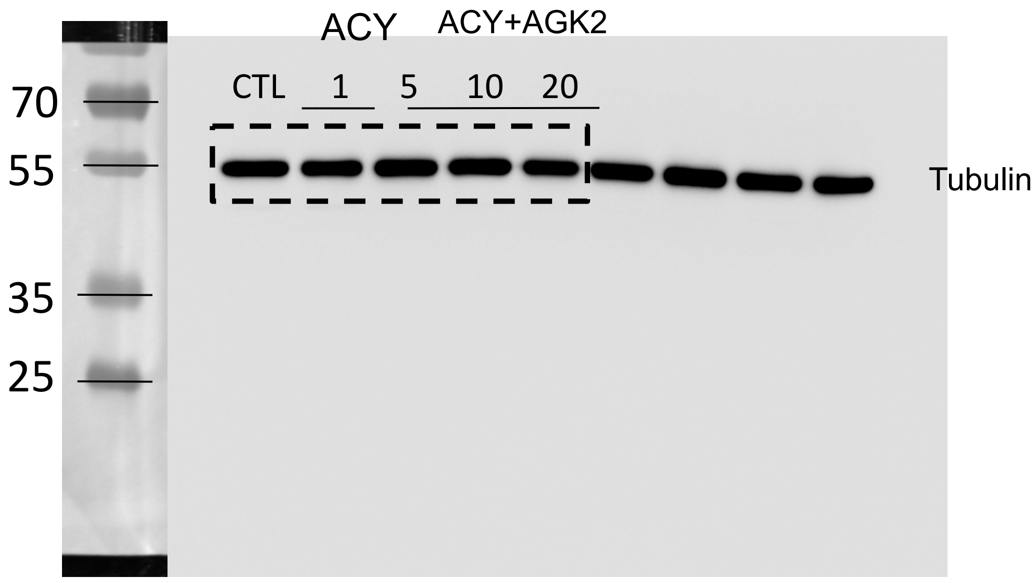

Supplemenatry Table 1

Genes	Primers
HDAC6-F	GCCTCAATCACTGAGACCATCC
HDAC6-R	GGTGCCTTCTTGGTGACCAACT
CTTN-F	TGTCCTCTGCCTACCAGAAGA
CTTN-R	CCTGCTCTTTCTCCTTAGCGA
aTAT-F	GGCGAGAACTCTTCCAGTAT
aTAT-R	TTGTTCACCTGTGGGACT
Sirt1-F	GGGAATCCAAAGGATAATTCAGTGT
Sirt1-R	CCTCGTACAGCTTCACAGTCAACT
Sirt2-F	AGCGGCTCCTCCCTCAGA
Sirt2-R	AGACCCAGGCAGGGAAGGT
HDAC1-F	GGTCCAAATGCAGGCGATTCCT
HDAC1-R	TCGGAGAACTCTTCCTCACAGG
HDAC2-F	CTCATGCACCTGGTGTCCAGAT
HDAC2-R	GCTATCCGCTTGTCTGATGCTC
HDAC3-F	TGATCGTCTTCAAGCCATACCA
HDAC3-R	TGTGTAACGCGAGCAGAACT
HDAC4-F	AGGTGAAGCAGGAGCCCATTGA
HDAC4-R	GGTAGTTCCTCAGCTGGTGGAT
HDAC5-F	CGCTGAGAATGGCTTTACTGGC
HDAC5-R	GTGTAGAGGCTGAACTGGTTGG
HDAC7-F	TCCTGGCACAGCGGATGTTTGT
HDAC7-R	TGAAGGCGAGGTCAGTGACACT
HDAC8-F	TGTGCTGGAAATCACGCCAAGC
HDAC8-R	ACCACTCCTCAGCTCTGGAAAC
HDAC9-F	TCTCGTCTCCAGGACTCACTCT
HDAC9-R	GCACTGGTGTTTCAGCATCAAGG
HDAC10-F	GAGGAGTCTGTGGCTGAACATC
HDAC10-R	CCAAGATGCAGCTCAGGAAACC
HDAC11-F	CTTCTGTGCCTATGCGGACATC
HDAC11-R	GAAGTCTCGCTCATGCCCATTG
